# Climate-induced variations in global wildfire danger from 1979 to 2013

**DOI:** 10.1038/ncomms8537

**Published:** 2015-07-14

**Authors:** W. Matt Jolly, Mark A. Cochrane, Patrick H. Freeborn, Zachary A. Holden, Timothy J. Brown, Grant J. Williamson, David M. J. S. Bowman

**Affiliations:** 1 US Forest Service, Rocky Mountain Research Station, Fire Sciences Laboratory, 5775 Highway 10 West, Missoula, Montana 59803, USA; 2 Geospatial Sciences Center of Excellence (GSCE), South Dakota State University, 1021 Medary Avenue, Wecota Hall, Box 506B, Brookings, South Dakota 57007, USA; 3 US Forest Service Region 1, 200 East Broadway Street, Missoula, Montana 59802, USA; 4 Desert Research Institute (DRI), Western Regional Climate Center, 2215 Raggio Parkway, Reno, Nevada 89512-1095, USA; 5 School of Biological Sciences, The University of Tasmania, Private Bag 55, Hobart, Tasmania 7001, Australia

## Abstract

Climate strongly influences global wildfire activity, and recent wildfire surges may signal fire weather-induced pyrogeographic shifts. Here we use three daily global climate data sets and three fire danger indices to develop a simple annual metric of fire weather season length, and map spatio-temporal trends from 1979 to 2013. We show that fire weather seasons have lengthened across 29.6 million km^2^ (25.3%) of the Earth's vegetated surface, resulting in an 18.7% increase in global mean fire weather season length. We also show a doubling (108.1% increase) of global burnable area affected by long fire weather seasons (>1.0 *σ* above the historical mean) and an increased global frequency of long fire weather seasons across 62.4 million km^2^ (53.4%) during the second half of the study period. If these fire weather changes are coupled with ignition sources and available fuel, they could markedly impact global ecosystems, societies, economies and climate.

## Abstract (web summary)

Global wildfires can have severe societal implications and economic cost and have been strongly linked to climate. Here, the authors analyse daily global wildfire trends and show that, during the past 35 years, wildfire season length has increased by 18.7% over more than a quarter of the Earth's surface.

## Graphical abstract

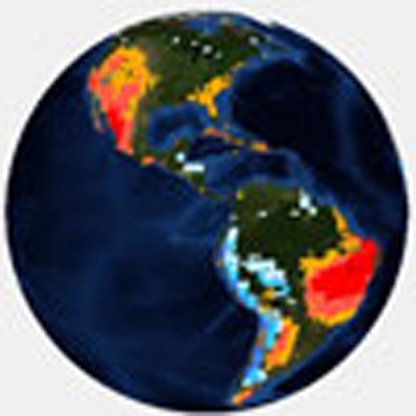

Wildfires play a pivotal, dynamic role in terrestrial and atmospheric systems^1^. Global annual burned area estimates approach 350 MHa per year^2^, and annual pyrogenic CO_2_ emissions can exceed 50% of fossil fuel combustion emissions^3^,^4^,^5^. Fires play an essential ecological role in flammable ecosystems: some are managed to clear forests, promote grazing and establish plants^6^, while others are suppressed to protect human lives and property, regardless of how they ignite. Recently, there has been a surge of extremely destructive fires with corresponding social disruptions and substantial economic costs. Over the last decade, annual wildfire suppression costs on US federal lands exceeded $1.7B US dollars^7^ and $1B US dollars in Canada^8^. When all components are considered, including preparedness/suppression costs and economic losses, these total costs are substantially higher. In Australia in 2005, total wildfire costs were estimated at nearly $9.4B US dollars or 1.3% of their Gross Domestic Product^9^. Therefore, the driving factors of contemporary wildfire activity changes must be understood to ensure that wildfires are effectively managed to promote healthy ecosystems while minimizing negative socio-economic impacts.

Wildfires occur at the intersection of dry weather, available fuel and ignition sources^10^. Weather is the most variable and largest driver of regional burned area^11^,^12^,^13^,^14^. Temperature, relative humidity, precipitation and wind speed independently influence wildland fire spread rates and intensities, and the alignment of multiple weather extremes, such as the co-occurrence of hot, dry and windy conditions leads to the most severe fires^15^. Global temperatures have increased by ∼0.2 °C per decade over the last three decades^16^, possibly leading to an acceleration of the global water cycle with more intense rainfall events^17^, more severe and widespread droughts^18^ (despite drought frequencies appearing unchanged^19^) and regional humidity variations^20^. Regional droughts are also tightly coupled to sea surface temperature variations^21^, and regional water availability variations can explain a significant proportion of the variations in burned area^22^. Climatic changes are implicated in global fire variations^23^ and are expected to increase fire season severity over the coming decades^24^. While several studies have examined climate-induced regional and circumboreal trends in fire danger^25^,^26^,^27^,^28^,^29^,^30^, a comprehensive global assessment of the interactions of recent climatic changes that lead to an expansion or contraction of fire seasons is lacking.

Landscape-scale fire behaviour is determined by local weather conditions, but biome-level wildfire potential is more appropriately associated with fire danger indices, which are representative of daily synoptic weather patterns^11^. Independent fire danger indices have been developed and applied for different regions worldwide. All are based on daily surface weather variables that are related to the ignitability, spread rate and the control difficulty of an initiating wildland fire^31^ and they are closely related to the magnitude and extent of fire activity^32^,^33^. Furthermore, these indices capture changes in fuel (live and dead plant material) moistures, and thus scale to fuel consumption^34^ and pyrogenic emission production^5^.

Here we present an analysis of daily global fire weather trends from 1979 to 2013 based on three sub-daily global meteorological data sets (the National Center for Environmental Prediction (NCEP) Reanalysis, NCEP-DOE Reanalysis II and the European Centre for Medium-Range Weather Forecasts (ECMWF) Interim Reanalysis)^35^,^36^,^37^ between ∼0.75° and 2.0° grid cell resolution. We use these data to calculate the US Burning Index^38^, the Canadian Fire Weather Index^39^ and the Australian (or McArthur) Forest Fire Danger Index^40^. Daily fire danger indices (normalized to a common scale and resampled to a common resolution) were used to derive a fire weather season length, defined as the number of days each year when fire danger is above half its value range, for each year in each grid cell using a technique commonly used to determine growing season length from satellite-derived vegetation indices^41^,^42^. Because climate studies using multi-model ensembles are generally superior to single model approaches^43^, all nine fire weather season lengths for each location were averaged into an ensemble mean fire weather season length, hereafter referred to as ‘Fire Weather Season Length' (See Supplementary Methods). This metric was examined to identify global and regional patterns in fire weather season length changes as well as changes in the frequency of, and the area affected by, long fire weather seasons (defined as >1.0 *σ* above historical mean) over the last 35 years.

## Results

### Global fire weather trends

**Figure 1:**
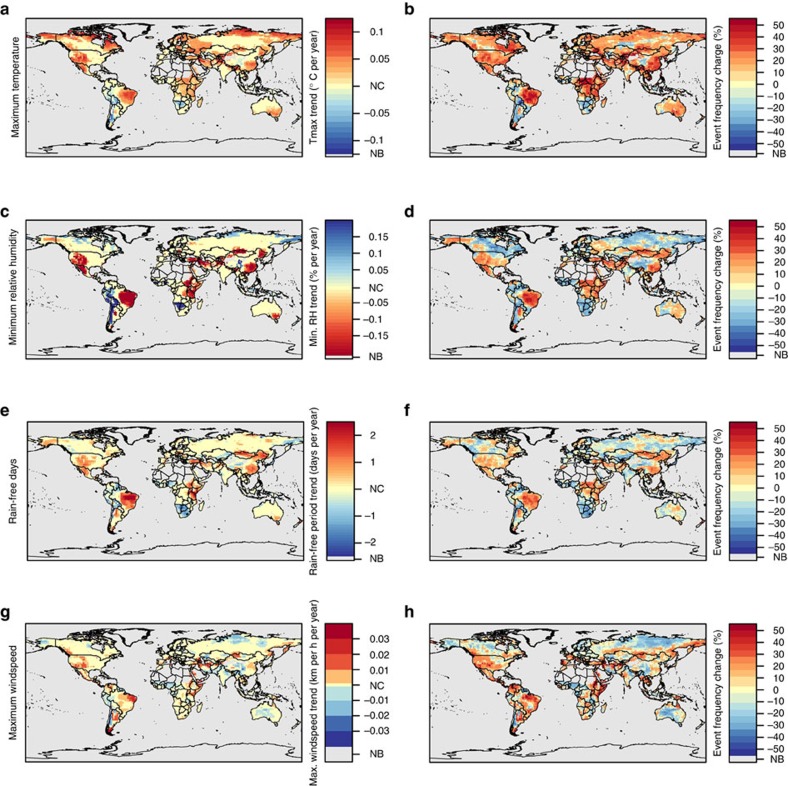
Long-term trends and changes in anomalous event frequency of maximum temperature, minimum relative humidity, annual rain-free days and maximum wind speed. **a**,**c**,**e**,and,**g** show areas with significant trends in annual fire weather variables. **b**,**d**,**f** and **h** show the change in frequency of the number of years with anomalous mean annual weather conditions (>1σ above historical mean) from 1996 to 2013 compared with the number of anomalies observed from 1979 to 1996. Areas with little or no burnable vegetation are shown in grey (NB) and NC indicates areas with no significant change. Red areas indicate locations where fire weather conditions are becoming increasingly more severe or anomalously severe weather events are becoming more frequent, while blue areas indicate locations where climatic influences on fire potential are lessening or weather events are becoming less frequent.

Ensemble mean annual maximum temperature across the vegetated land surface increased by 0.184 °C per decade from 1979 to 2013 (Supplementary Table 1). This global trend is comparable to the global land temperature trends reported by the Intergovernmental Panel on Climate Change of 0.268, 0.315, 0.188 and 0.203 K per decade^4^. Mean annual global vegetated surface maximum temperature was significantly correlated to the Goddard Institute of Space Studies (GISS) land-surface temperature anomalies (*ρ*=0.88, *P*<0.001)^16^. The amount of area witnessing unusually hot years, where maximum temperature was more than 1 s.d. from the mean, also showed a significant increase of 6.3% per decade (Supplementary Table 1). Mean annual minimum relative humidity showed a weak but significant trend of −0.127% per decade but showed no significant changes in affected area. There were no significant trends in mean annual total precipitation or total precipitation affected area but we did observe a significant increase in mean annual rain-free days, where the mean number of dry days increased by 1.31 days per decade and the global area affected by anomalously dry years significantly increased by 1.6% per decade. Despite an absence of global trends in some key meteorological variables, there were distinct regional annual climate trends and changes in anomalous weather event affected area frequency (Fig. 1).

### Global fire weather season length trends

**Figure 2:**
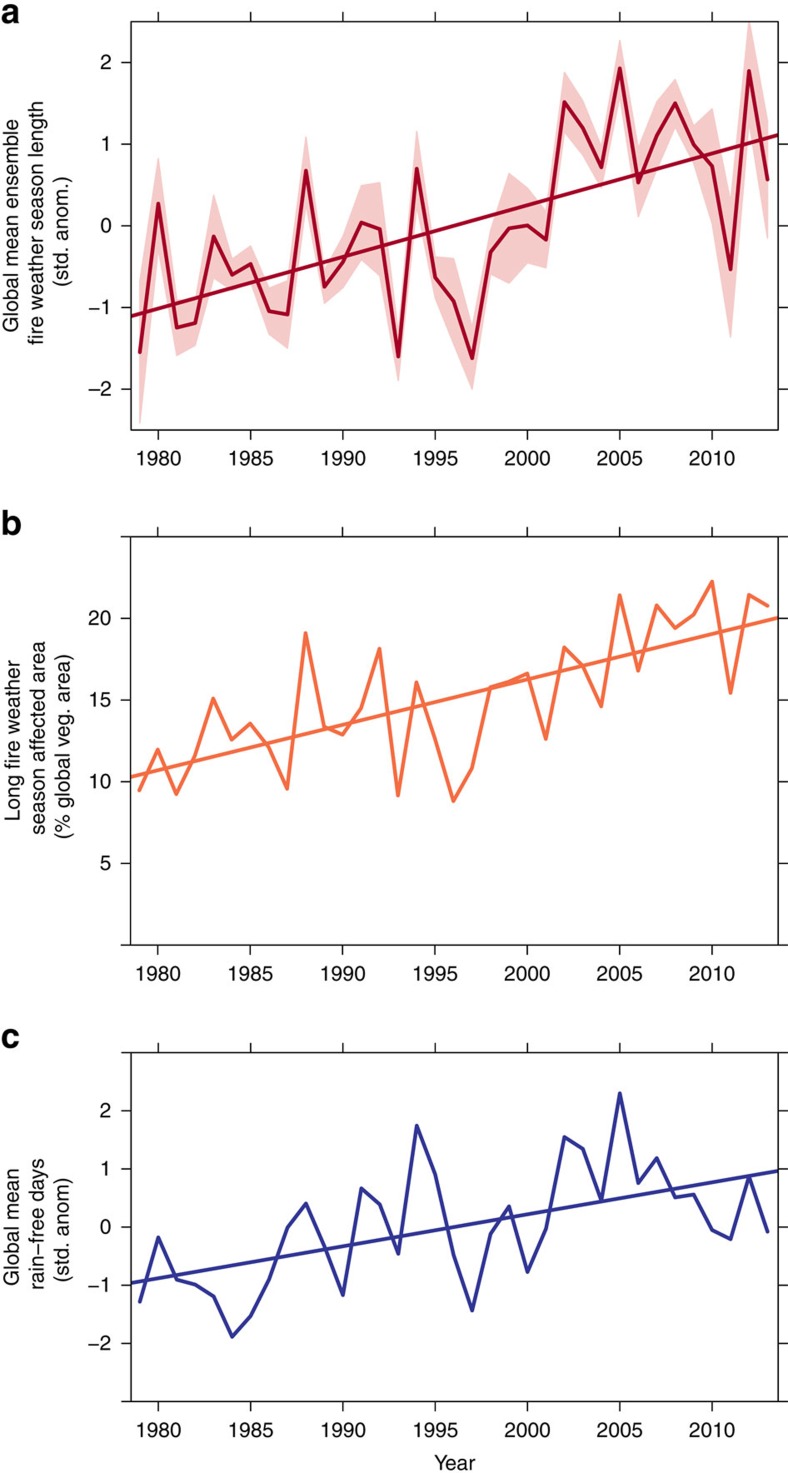
Trends in global fire weather season length metrics and rain-free days. (**a**) Changes in the global mean fire weather season length from 1979 to 2013 with 95% confidence limits between ensemble members (shaded area). (**b**) Total global annual area affected by long fire weather seasons (>1σ of historical mean). (**c**) Inter-annual variations in the standard anomalies of global mean rain-free days. Global mean rain-free days accounted for 49.7% of the variation in global fire weather season length and 33.8% of the variation in global long fire weather season affected area.

**Figure 3:**
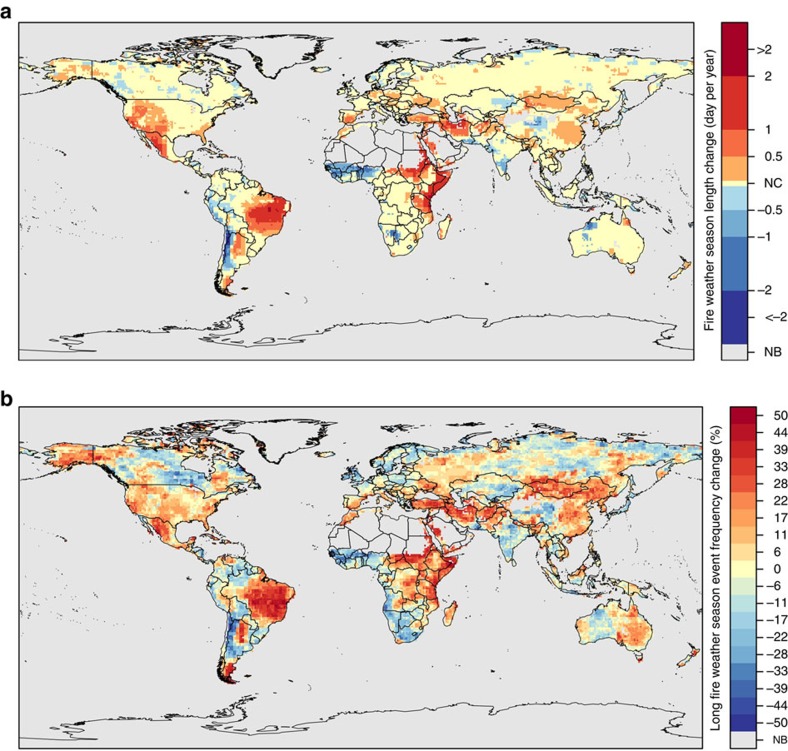
Global patterns of fire weather season length changes from 1979 to 2013. **a** shows areas with significant trends in fire weather season length from 1979 to 2013. **b** shows regions that have experienced changes in the frequency of long fire weather seasons (>1σ above historical mean) during the second half of the study period (1996–2013) compared with the number of events observed during the first half (1979–1996). Areas with little or no burnable vegetation are shown in grey (NB) and NC indicates areas with no significant change. Reds indicate areas where fire weather seasons have lengthened or long fire weather seasons have become more frequent. Blues indicate areas where fire weather seasons have shortened or long fire weather seasons have become less frequent.

**Figure 4:**
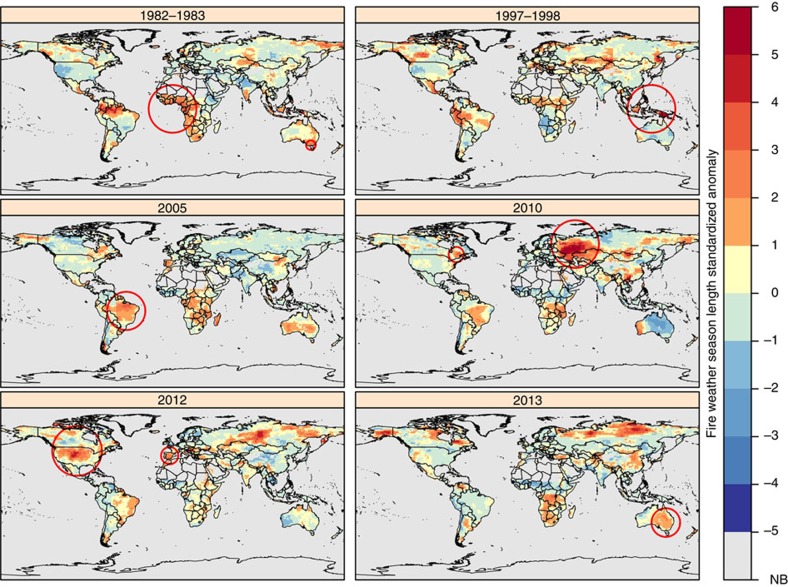
Examples of fire weather season length standardized anomalies during significant global fire events. Red colours indicate areas where fire weather season anomalies are >1 s.d. from the mean, while blue areas indicate shorter-than-normal fire weather season lengths. Areas with little or no burnable vegetation are shown in grey (NB). Red circles denote regions with significant fire activity during that time period. El Niño periods often span multiple calendar years (for example, 1982–1983), so in these cases, the maximum anomaly of either year was mapped above. Maps of all years (1979–2013) are included as Supplementary Figs 1–4.

Even more distinct patterns and trends in annual climate anomalies emerged when the meteorological data were used to calculate fire danger indices and then combined into metrics characterizing the fire weather season length and long fire weather season affected areas. Globally, fire weather season length increased by 18.7% from 1979 to 2013 (Fig. 2a), with statistically significant increases observed across 25.3% (29.6 M km^2^) of the global vegetated area and decreases in only 10.7% (12.5 M km^2^) (Fig. 3a). Long fire weather season affected area, defined as the total global area observing fire weather seasons >1 s.d. from the mean, has increased by 3.1% per year from 1979 to 2013, leading to a 108.1% increase in global long fire weather season affected area (Fig. 2b). The frequency of long fire weather seasons increased across 53.4% of the global vegetated area (62.4M km^2^) as observed between 1996 and 2013, compared with 1979–1996, with decreased frequency only observed across 34.6% (40.4 M km^2^) (Fig. 3b). Since 1979, there have been 6 years, all in the last decade, where >20% of the global vegetated area has been affected by long fire weather seasons (2005, 2007, 2009, 2010, 2012 and 2013). Both fire weather season metrics were strongly correlated with the global mean annual number of days without wetting rainfall (>0.1 mm) (Fig. 2c), where global mean rain-free days accounted for 49.7% of the variation in global fire weather season length and 33.8% of the variation in global long fire weather season affected area. Annual fire weather season length anomaly maps for a subset of known severe fire years are presented in Fig. 4 and anomalies for all years are presented in Supplementary Figs 1–4 and annual ensemble-mean anomaly data are available as Supplementary Data 1.

Fire weather season length and long fire weather season affected area increased significantly across all continents except Australia (Table 1). Globally, most biomes showed significant increases in fire weather season metrics with the exceptions of temperate and montane grasslands, savannas and shrublands and boreal forests/taiga and tundra (Table 2). The strongest trends were observed in tropical and subtropical grasslands, savannas and shrublands. Continent × biome trends indicate that regional variations in fire weather season metrics were much stronger than global trends (Table 3).

### Comparisons to country-wide reported burned area

Inter-annual variations in mean US fire weather season length were significantly correlated with variation in annual burned area reported by the US National Interagency Fire Center^44^ over the full time series from 1979 to 2013 and also from 1992 to 2013, when fire occurrence data quality was highest^45^ (*ρ*=0.679 and 0.683, respectively, *P*<0.001). Further, long fire weather season affected area was also significantly correlated to the burnt area variations of the full and limited time series (*ρ*=0.663 and 0.715, respectively, *P*<0.001) (Table 4 and Supplementary Fig. 5). Inter-annual variations in mean fire weather season length were also significantly correlated to inter-annual burned area variations from 1980 to 2013 across Spain, Portugal, France, Italy, Greece and Latvia (Table 4), and inter-annual variations in long fire weather season affected area were significantly correlated with burned area for Spain, Portugal, Italy and Latvia (Table 4 and Supplementary Figs 6–8). Both mean fire weather season length and long fire weather season affected area, constrained to only boreal forests where most Canadian fires occur, were only weakly correlated to burned area across Canadian forests (*ρ*=0.3 and 0.324, respectively, *P*<0.1) (Table 4 and Supplementary Fig. 5).

### Comparisons to global land carbon uptake

Variation in fire danger metrics have been shown to be closely related to fuel consumption^34^, and fire activity is generally inversely correlated with vegetation productivity^47^. Likewise, fire weather season length and long fire weather season affected area were significantly correlated with global net land carbon flux calculated from an analysis of the global carbon budget from 1979 to 2012 (ref. 48) (See Supplementary Methods) (*ρ*=−0.38 and *ρ*=−0.48, *n*=33, *P*<0.05 and *P*<0.01, respectively, Supplementary Fig. 9). In addition, when correlations were constrained to the time period that satellite burned area observations were available from the Moderate Resolution Imaging Spectroradiometer (MODIS) (2001-2012), and thus where estimates of land-use change carbon emissions were more certain^2^, correlations between fire weather season length, long fire season affected area and net land carbon fluxes increased substantially to *ρ*=−0.797 and *ρ*=−0.825, respectively, *n*=12, *P*<0.01). The highest correlations between the net land carbon flux and continental biome mean fire weather season metrics were observed in the tropical and subtropical forests, grasslands and savannas and xeric shrublands of South America where regional fire weather season length metrics accounted for between 15.7 and 29.7% of the variations in global net land carbon flux (Table 5).

## Discussion

Fire weather season length and long fire weather season affected area significantly increased across all vegetated continents except Australia. For example, significant fire weather season lengthening has occurred throughout much of Africa, particularly the subtropical grasslands and savannas of the eastern half of the continent (Fig. 3) and across Africa's Mediterranean forests, woodlands and scrub. Persistent fire weather season length increases in ecosystems such as South Africa's Mediterranean fynbos could lead to more frequent severe burning conditions and more area burned, shortening fire return intervals and threatening these biodiversity-rich shrublands^49^. Recent climatic changes have mitigated wildland fire potential in some regions of Africa since 1979. For example, drought during the early 1980s (ref. 50) lengthened fire weather seasons in the West African subtropics. Drought conditions have subsequently subsided, leading to a steady contraction of the fire weather season lengths across much of West Africa (Fig. 3).

Our ensemble fire weather season length metric captured important wildfire events throughout Eurasia such as the Indonesian fires of 1997–98 where peat fires, following an El Niño-induced drought, released carbon equivalent to 13–40% of the global fossil fuel emissions from only 1.4% of the global vegetated land area (Fig. 4, 1997–1998)^46^ and the heatwave over Western Russia in 2010 (Fig. 4, 2010) that led to its worst fire season in recorded history and triggered extreme air pollution in Moscow^51^. Our metric also revealed similar impacts across Eastern Canada in 2010, where high temperatures and significant water deficits led to large wildfires (Fig. 4, 2010)^52^. European Mediterranean forests were also identified as being susceptible to significant changes: the inner-quartile range of fire weather season length trends indicate a lengthening of 12 to 19 days, with a maximum increase of nearly a month (29 days) from 1979 to 2013 (Table 6). This is consistent with a lengthening of the fire weather season in Spain during 2012 (Fig. 4, 2012) where fires burned more area than any year in the previous decade^49^.

Over the last several decades, the US has witnessed a marked increase in large wildfire frequency and duration with the greatest increases observed in the temperate coniferous forests of the Northern Rocky Mountains^53^,^54^. These trends are widely attributed to shifts towards earlier snowmelt timing^54^, though fire activity in the desert Southwest has also been attributed to warming-driven increases in vapour pressure deficit^55^ and variability in the timing of spring precipitation^56^. Our results extend these findings by demonstrating that areas with the most significant change in fire weather season length occur where not only temperature but also changes in humidity, length of rain-free intervals and wind speeds are most pronounced. In 2012, for example, longer-than-normal fire weather seasons across an unprecedented 47.4% of the vegetated area of the US (Fig. 4, 2012) culminated in a near-record setting ∼3.8 MHa of burned area. In addition, our results show significant fire weather season lengthening throughout much of the Eastern US Coastal Plains. Over the last decade, this region has witnessed a marked uptick in wildfires and a group of large fires in Okefenokee National Wildlife Refuge, the Osceola National Forest and adjacent lands burned ∼243 KHa in 2007 (ref. 57), one of those fires ranked as the twelfth largest fire in the US history.

Fire weather season length and long fire weather season affected area were only weakly correlated to Canadian boreal forest burned area (Table 4). This may be caused by averaging across the large zonal and meridional climatic gradients across the country. Future work should examine relationships between provincial fire activity and fire weather season metrics. In addition, our fire weather season length metric captures variations in the number of days each year that fires are likely to burn, but it does not account for inter-annual variations in fire season severity. Further work should consider both a lengthening fire season and an increase in within-season fire weather severity as causal mechanisms of burned area variations.

South America's tropical and subtropical forests, grasslands and savannas have experienced tremendous fire weather season length changes, with a median increase of 33 days over the last 35 years (Fig. 3a and Table 6 ). Our metric captured a rare drought in the Amazon in 2005 that prompted long fire weather seasons, leading to a dramatic increase in basin-wide fire activity^58^ (Fig. 4, 2005). Longer fire seasons prolong conducive burning conditions, potentially expanding areas susceptible to escaped deforestation fires^59^ and subsequently strengthening feedbacks in regional climate-fire dynamics^60^.

In contrast to all other continents in our analysis, Australia showed no significant trends in biome-level fire season length, but we identified regional increases in the frequency of anomalously long fire weather seasons, especially from 1996 to 2013 (Fig. 3b). Our analysis also captures climatic droughts that contributed to the Ash Wednesday fires in Victoria (1983) (Fig. 4, 1982–1983), the Canberra bushfires (2003) (Supplementary Fig. 3), the Black Saturday fires (2009) (Supplementary Fig. 4) and the widespread heatwave across Australia in the summer of 2012–2013 that promoted long fire weather seasons and intense bushfire activity across most of the Eastern half of the country (Fig. 4, 2013). Australian fires seasons are dominated by periods of benign weather followed by years with extreme fire weather conditions. Such high inter-annual variability reflects climate modes, such as the El Niño Southern Oscillation (ENSO) and the Indian Ocean Dipole, which can heavily influence inter-annual rainfall variations and subsequently affect wildfire potential throughout Australia^61^,^62^.

Fire weather season length imperfectly scales with actual fire activity because fires may not be ignited, there may be no available fuel, or they may be suppressed by humans. Nonetheless, our global fire weather season length metrics were significantly correlated to global net land carbon flux. These correlations were negative, suggesting that when average fire weather seasons are longer-than-normal or when long seasons impacted more global burnable area, net global terrestrial carbon uptake is reduced. Generally, low correlations between fire weather season length and global land carbon uptake are to be expected because wildfires represent a small proportion of the total land carbon flux. However, if our fire season metrics were combined with other metrics of global land carbon uptake that have been produced by others^63^,^64^, they may improve our ability to assess the cumulative impacts of climatic changes on terrestrial carbon fluxes. Correlations between global net land carbon flux and continental-scale, biome mean fire weather season length metrics were highest across South American tropical and subtropical forests, savannas and grasslands and xeric shrublands (Table 5), highlighting that the strongest coupling between fire weather and global carbon emissions is occurring in an area of intense land-use pressure.

Global fire regimes are the combined results of available fuel, ignition sources and conducive fire weather^10^. As such, leveraging well-established fire danger indices to explore changes in global wildfire weather only capture part of the potential variations in global pyrogeography. Regionally, our documented fire weather changes may not appreciably alter fire regimes if fires are not ignited or if there is no fuel. However, we observed an overall lengthening of the number of days each year that wildfires may burn across more than a quarter of the Earth's vegetated surface and these fire weather changes could manifest themselves as a positive feedback to global atmospheric carbon accumulations if all the requirements for wildfires are present. In addition, this study may improve our ability to explore the complex drivers of global fire activity by isolating the climate-induced variations in fire potential from changes in either fuel availability or human and nature-caused ignitions, which lead to the realized burned area and subsequent fire emissions. In summary, we have shown that combined surface weather changes over the last three and a half decades have promoted global wildfire weather season lengthening. If these trends continue, increased wildfire potential may have pronounced global socio-economic, ecological and climate system impacts.

## Methods

### Meteorological data

Three global reanalysis projects provided gridded, sub-daily surface meteorological data from 1979 to 2013. Two data sets at ∼210 km spatial resolution were obtained from NCEP, including the Reanalysis I^36^ and the DOE Reanalysis II data sets^37^. Six-hourly data fields for 2 m maximum temperature, minimum temperature, specific humidity, surface pressure, precipitation rate, water equivalent of actual snow depth and 10 m *U* and *V* wind components were summarized to daily data (Supplementary Table 2). We assumed that diurnal variations in actual vapour pressure are small and thus daily mean actual vapour pressure was calculated from the NCEP data using mean daily specific humidity and surface pressure^65^, and saturation vapour pressure was calculated from daily maximum and minimum temperature to calculate daily maximum and minimum relative humidity. In addition, we used the ECMWF ERA Interim Reanalysis^35^. This data set is similar to NCEP's but at a higher spatial and temporal resolution (∼78 km resolution). We extracted 3-hourly 2 m air temperature, dewpoint temperature, surface total precipitation, and 10 m *U* and *V* wind components using the ECMWF GRIdded Binary Application Programming Interface (GRIB-API) and used them to derive daily maximum and minimum temperature, maximum and minimum relative humidity, maximum wind and total daily precipitation amount and daily precipitation duration (Supplementary Table 3). Daily maximum and minimum relative humidity were calculated using mean daily dewpoint temperature and minimum and maximum daily 2 m air temperature, respectively.

### Surface meteorological data analysis

Trends in ensemble mean annual values were analysed for five climate variables that are important to wildland fire potential: mean maximum daily temperature, mean minimum daily relative humidity, total precipitation, rain-free days and mean maximum 10 m wind speed. Annual means or totals for each pixel were averaged across all three reanalysis data sets to produce an ensemble mean value. Two types of analysis were performed on each ensemble climate variable. First, we examined areas that showed long-term trends in mean annual weather conditions. All trends were evaluated using Mann–Kendall trend tests, following a four-step process to reduce the effects of serial autocorrelation on significance tests^66^ (see Supplemental Methods). Second, we examined the change in frequency of occurrence of unusually hot, dry or windy conditions by comparing the number of years that maximum temperature, rain-free days or wind speed was >1 s.d. above the mean or when minimum relative humidity was <1 s.d. from the mean in 1996–2013, as compared with the number of similar events observed in 1979–1996. One year, 1996, overlaps each period to provide 18 years in each period. Further, all data sets were masked using the vegetated (burnable) land area defined by a global landcover data set developed from AVHRR satellite data^67^. Mean trends and event frequency changes were then displayed spatially (Fig. 1) and summarized to average global values (Supplementary Table 1).

## Additional information

**How to cite this article:** Jolly, W. M. *et al.* Climate-induced variations in global wildfire danger from 1979 to 2013. *Nat. Commun.* 6:7537 doi: 10.1038/ncomms8537 (2015).

## Supplementary Material

Supplementary Information Supplementary Figures 1-11, Supplementary Table 1-4, Supplementary Methods and Supplementary References

Supplementary Data 1 Annual Ensemble Fire Weather Season Length Standardized-Anomalies from 1979-2013 rasters in GeoTIFF format.

## Figures and Tables

**Table 1:** Continental trends in fire weather season length and long fire weather season affected area.

**Continent**	**Mean fire weather season length trend (days per year)**	**Long fire weather season affected area trend (% glob. veg. area per year)**
Africa	0.1542**	NS
Eurasia	0.0475***	0.0973***
Australia/New Zealand	NS	NS
North America	0.1519***	0.0726**
South America	0.2249**	0.0545**

NS, not significant.

Results of non-parametric tests examining temporal trends in the continental-mean fire weather season length and long fire weather season affected area from 1979 to 2013. (***P<0.01, **P<0.05, *P<0.1). Slopes were estimated using the Theil–Sen non-parametric trend slope estimator and significance tests were performed using the Mann–Kendall trend test following a four-step approach to reduce the effects of serial autocorrelation on significance tests^66^.

**Table 2:** Global biome trends in fire weather season length and long fire weather season affected area.

**Biome**	**Mean fire weather season length trend (days per year)**	**Long fire weather season affected area trend (% glob. veg. area per year)**
Tropical forests	0.089**	NS
Temperate broadleaf and mixed forests	0.103***	0.0464***
Temperate conifer forests	0.184***	0.0150***
Tropical and subtropical grasslands, savannas and shrublands	0.220***	0.06278**
Temperate and montane grasslands, savannas and shrublands	NS	0.0255**
Boreal forests/taiga and tundra	NS	NS
Mediterranean forests, woodlands and scrub	0.159**	0.0105***
Xeric shrublands	0.151***	0.0535***

NS, not significant.

Results of non-parametric tests examining temporal trends in the biome mean fire weather season length and long fire weather season affected area. (***P<0.01, **P<0.05, *P<0.1). Slopes were estimated using the Theil–Sen non-parametric trend slope estimator and significance tests were performed using the Mann–Kendall trend test following a four-step approach to reduce the effects of serial autocorrelation on significance tests^66^.

**Table 3:** Continental × biome trends in fire weather season length and long fire weather season affected area.

**Continent**	**Biome**	**Mean fire weather season length (EFWSL)** **trend (days per year)**	**Long fire weather season affected area (ALFWS) affected area trend (% area per year)**
Africa	Tropical forest	0.1321**	NS
	Tropical and subtropical grasslands, savannas and shrublands	0.1899**	0.3181*
	Temperate and montane grasslands, savannas and shrublands	NS	NS
	Mediterranean forests, woodlands and scrub	0.2292**	0.5642*
	Xeric shrublands	NS	NS
Eurasia	Tropical forest	−0.0833*	NS
	Temperate broadleaf forests	0.1362***	0.517***
	Temperate conifer forests	0.1295***	0.6211***
	Tropical and subtropical grasslands, savannas and shrublands	NS	NS
	Temperate and montane grasslands, savannas and shrublands	0.0875**	0.3798**
	Boreal forests/taiga and tundra	NS	NS
	Mediterranean forests, woodlands and scrub	0.2877***	0.7607***
	Xeric shrublands	0.1435**	0.4243***
Australia/New Zealand	Tropical forest	NS	NS
	Temperate broadleaf forests	NS	NS
	Tropical and subtropical grasslands, savannas and shrublands	NS	NS
	Temperate and montane grasslands, savannas and shrublands	NS	NS
	Mediterranean forests, woodlands and scrub	NS	NS
	Xeric shrublands	NS	NS
North America	Tropical forest	0.3297***	0.454**
	Temperate broadleaf forests	NS	NS
	Temperate conifer forests	0.184***	0.4215**
	Tropical and subtropical grasslands, savannas and shrublands	NS	NS
	Temperate and montane grasslands, savannas and shrublands	NS	NS
	Boreal forests/taiga and tundra	NS	NS
	Mediterranean forests, woodlands and scrub	NS	NS
	Xeric shrublands	0.4156***	0.6618**
South America	Tropical forest	0.1461**	NS
	Temperate broadleaf forests	−0.1143***	NS
	Tropical and subtropical grasslands, savannas and shrublands	0.5027***	0.9297***
	Temperate and montane grasslands, savannas and shrublands	−1.4452***	−1.1156,***
	Xeric shrublands	0.4307*	0.6813**

NS, not significant.

Results of non-parametric tests examining temporal trends in the continental-level, biome mean fire weather season length and long fire weather season affected area. Shaded areas indicate either an absence or a very low percentage cover of that biome on that continent. All area trends are expressed as the percentage change of the total area of each biome on each continent (***P<0.01, **P<0.05, *P<0.1). Slopes were estimated using the Theil–Sen non-parametric trend slope estimator and significance tests were performed using the Mann–Kendall trend test following a four-step approach to reduce the effects of serial autocorrelation on trend test^66^.

**Table 4:** Correlations between fire weather season length metrics and reported nationwide burned area.

**Country**	**Time period**	**Fire weather season length**	**Long fire weather season affected area**
Canada^68^	1979–2013	0.30* (34)	0.324*NS (34)
United States^44^	1979–2013	0.679*** (21)	0.663*** (21)
United States^44^	1992–2013	0.683*** (34)	0.715*** (34)
Spain^69^	1980–2013	0.703*** (33)	0.743*** (27)
Portugal^69^	1980–2013	0.659*** (33)	0.67*** (19)
Italy^69^	1980–2013	0.823*** (33)	0.772*** (27)
France^69^	1980–2013	0.469*** (33)	NS
Greece^69^	1980–2013	0.611*** (33)	NS
Latvia^69^	1980–2013	0.694*** (33)	0.845** (10)

NS, not significant.

Spearman's rank-order correlation between detrended fire weather season length, long fire weather season affected area and log-transformed national burned area data reported for two countries in North America and six countries in Europe (***P<0.01, **P<0.05, *P<0.1). Canadian burned area was limited to boreal forests where most of the burned area occurs^70^. Number of data points after detrending are reported in parentheses.

**Table 5:** Correlations between fire weather season length metrics and global net land carbon flux by continent and biome.

**Continent**	**Biome**	**Correlation between mean fire weather season length and global net land carbon flux**	**Correlation between long fire weather season affected area and global net land carbon flux**
Africa	Tropical forest	NS	NS
	Tropical and subtropical grasslands, savannas and shrublands	−0.358**	−0.431**
	Temperate and montane grasslands, savannas and shrublands	NS	NS
	Mediterranean forests, woodlands and scrub	NS	NS
	Xeric shrublands	NS	NS
Eurasia	Tropical forest	NS	NS
	Temperate broadleaf forests	NS	NS
	Temperate conifer forests	NS	−0.036
	Tropical and subtropical grasslands, savannas and shrublands	NS	NS
	Temperate and montane grasslands, savannas and shrublands	NS	NS
	Boreal forests/taiga and tundra	NS	NS
	Mediterranean forests, woodlands and scrub	NS	−0.377**
	Xeric shrublands	0.381**	0.467***
Australia/New Zealand	Tropical forest	−0.292*	NS
	Temperate broadleaf forests	NS	NS
	Tropical and subtropical grasslands, savannas and shrublands	NS	NS
	Temperate and montane grasslands, savannas and shrublands	NS	NS
	Mediterranean forests, woodlands and scrub	NS	−0.325*
	Xeric shrublands	NS	NS
North America	Tropical forest	NS	−0.299*
	Temperate broadleaf forests	NS	NS
	Temperate conifer forests	NS	NS
	Tropical and subtropical grasslands, savannas and shrublands	NS	0.341*
	Temperate and montane grasslands, savannas and shrublands	NS	NS
	Boreal forests/taiga and tundra	NS	NS
	Mediterranean forests, woodlands and scrub	NS	NS
	Xeric shrublands	NS	NS
South America	Tropical forest	−0.414**	−0.489***
	Temperate broadleaf forests	NS	−0.434**
	Tropical and subtropical grasslands, savannas and shrublands	−0.456***	−0.396**
	Temperate and montane grasslands, savannas and shrublands	NS	NS
	Xeric shrublands	−0.545***	−0.397**

NS, not significant.

Spearman's rank-order correlation coefficients between first-difference detrended global net land carbon flux and continental-level, biome mean fire weather season length and long fire weather season affected area (***P<0.01, **P<0.05, *P<0.1).

**Table 6:** Distribution of significant trends in fire weather season length and long fire weather season affected area by continent and biome.

**Continent**	**Biome**	**Min**	**1**^**st**^ **quarter**	**Median**	**3**^**rd**^ **quarter**	**Max**	***N***
Africa	Tropical forest	−1.33	−0.116	0.287	0.641	2.120	147
	Tropical and subtropical grasslands, savannas and shrublands	−1.790	0.668	0.597	1.11	2.22	853
	Temperate and montane grasslands, savannas and shrublands	−0.75	−0.333	0.254	0.448	1.76	30
	Mediterranean forests, woodlands and scrub	0.205	0.306	0.360	0.465	0.775	29
	Xeric shrublands	−1.55	−0.867	0.477	1.10	1.98	141
Eurasia	Tropical forest	−1.04	−0.295	0.0417	0.114	1.46	335
	Temperate broadleaf forests	0.257	0.106	0.161	0.271	2.02	642
	Temperate conifer forests	−0.318	0.0767	0.114	0.248	2.14	158
	Tropical and subtropical grasslands, savannas and shrublands	−0.578	0.059	0.0715	0.111	0.179	5
	Temperate and montane grasslands, savannas and shrublands	−1.14	0.0608	0.182	0.2590	1.62	659
	Boreal forests/taiga and tundra	−0.496	−0.136	−0.0519	0.063	0.251	711
	Mediterranean forests, woodlands and scrub	−0.285	0.336	0.414	0.542	0.83	123
	Xeric shrublands	−1.22	−0.252	0.363	0.665	2.15	563
Australia/New Zealand	Tropical forest	−0.537	−0.523	−0.429	−0.172	1.67	4
	Temperate broadleaf forests	0.0932	0.108	0.147	0.153	0.301	25
	Tropical and subtropical grasslands, savannas and shrublands	−1.34	−0.965	−0.633	0.49	0.968	51
	Temperate and montane grasslands, savannas and shrublands	0.0428	0.106	0.111	0.13	0.13	35
	Mediterranean forests, woodlands and scrub	−0.559	−0.278	0.181	0.315	0.341	9
	Xeric shrublands	−1.09	−0.703	−0.548	−0.291	0.439	62
North America	Tropical forest	−0.364	0.391	0.719	1.28	1.97	112
	Temperate broadleaf forests	−0.264	0.0492	0.105	0.17	0.228	66
	Temperate conifer forests	−0.222	0.0315	0.246	0.441	1.03	288
	Tropical and subtropical grasslands, savannas and shrublands	0.119	0.126	0.133	0.141	0.148	2
	Temperate and montane grasslands, savannas and shrublands	0.0469	0.261	0.322	0.464	0.813	120
	Boreal forests/taiga and tundra	−0.302	−0.10	0.0399	0.0731	0.264	809
	Mediterranean forests, woodlands and scrub	−0.823	0.624	0.761	0.893	1.410	16
	Xeric shrublands	0.162	0.433	0.56	0.681	1.210	287
South America	Tropical forest	−1.76	−0.0863	0.252	0.731	2.04	706
	Temperate broadleaf forests	−2.53	−0.557	0.0288	0.0957	0.275	36
	Tropical and subtropical grasslands, savannas and shrublands	−0.887	0.62	0.947	1.30	2.39	320
	Temperate and montane grasslands, savannas and shrublands	−2.64	−0.829	−0.375	0.302	0.966	258
	Xeric shrublands	−2.660	−0.588	1.440	1.650	2.070	112

Statistical summaries of all significant, pixel-based fire weather season length linear trends from 1979 to 2013 by biome for Africa and Eurasia. All values are trend coefficients in days per year, representing an increase or decrease in the fire weather season length. *N* represents the number of significant pixels in each continent × biome. Slopes were estimated using the Theil–Sen non-parametric trend slope estimator.
