# Supplementary material for: Climate-induced variations in global wildfire danger from 1979 to 2013
Source: Nat Commun. 2015 Jul 14;6:7537. doi: 10.1038/ncomms8537 (PMC4803474; doi:10.1038/ncomms8537)
Supplement: Supplementary Information — Supplementary Figures 1-11, Supplementary Table 1-4, Supplementary Methods and Supplementary References [file ncomms8537-s1.pdf]

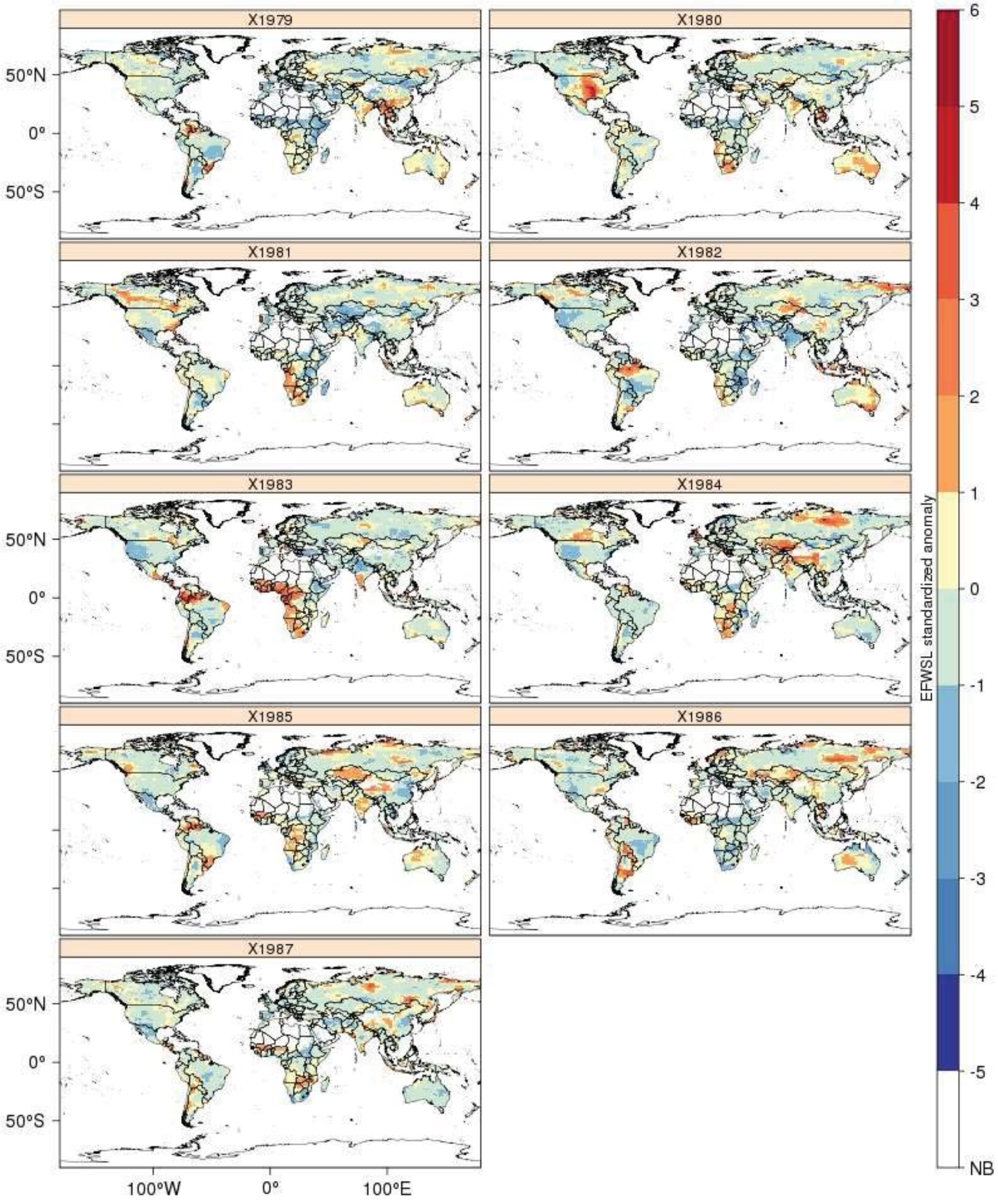

Supplementary Figure 1 – Maps of standardized anomalies of Fire Weather Season Length (Equation 5) from 1979-1987.

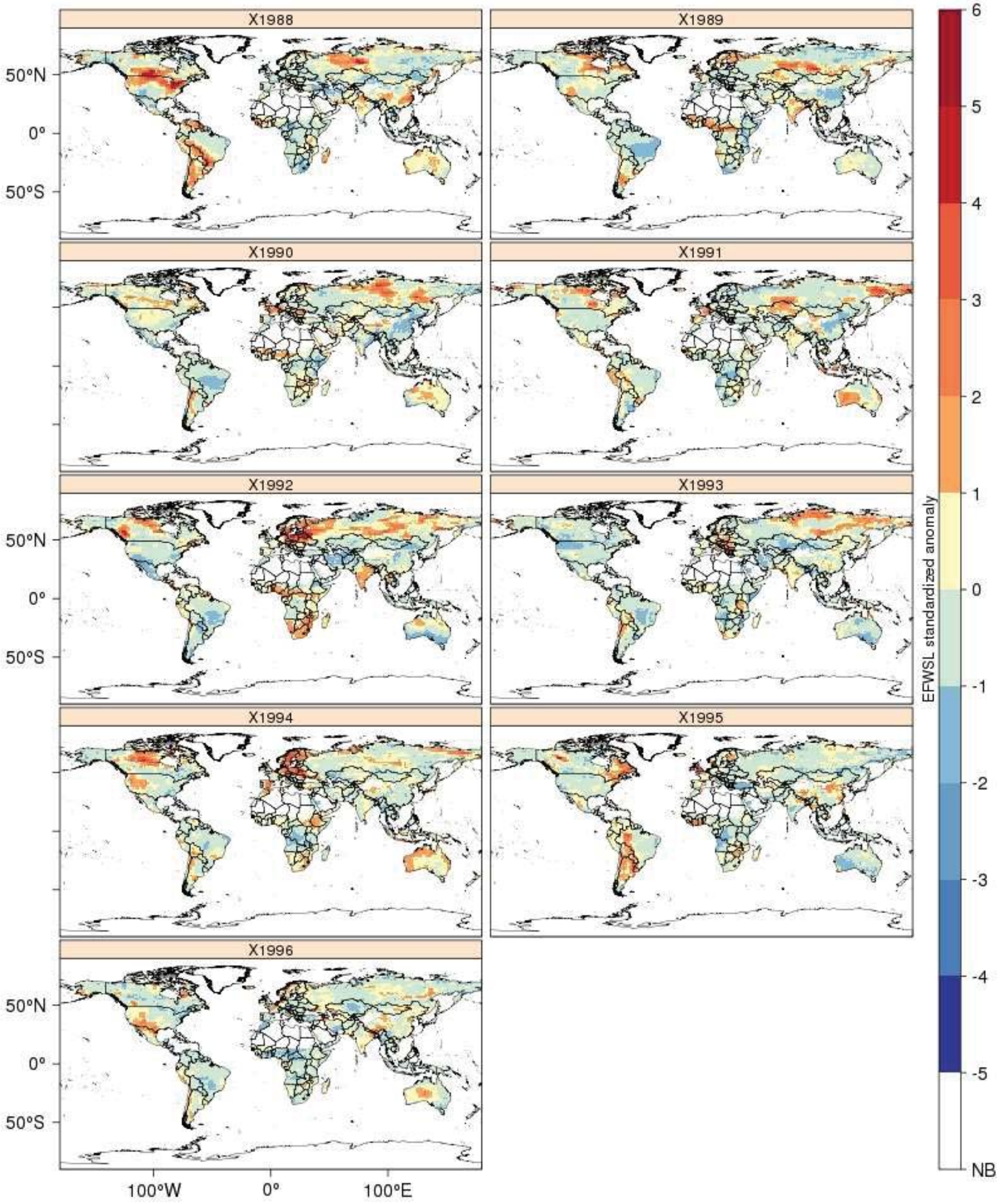

Supplementary Figure 2 – Maps of standardized anomalies of Fire Weather Season Length (Equation 5) from 1988-1996.

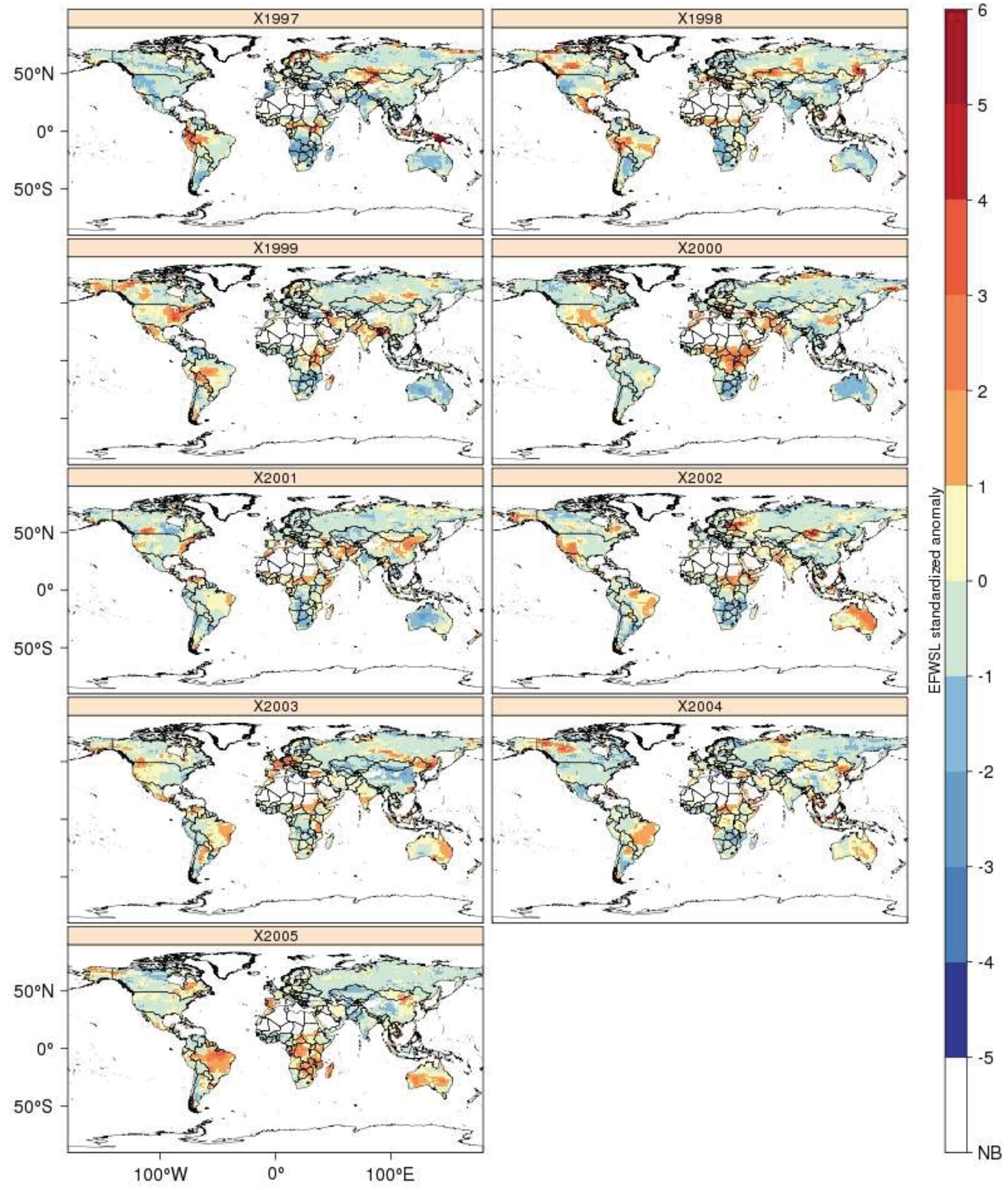

Supplementary Figure 3 – Maps of standardized anomalies of Fire Weather Season Length (Equation 5) from 1997-2005.

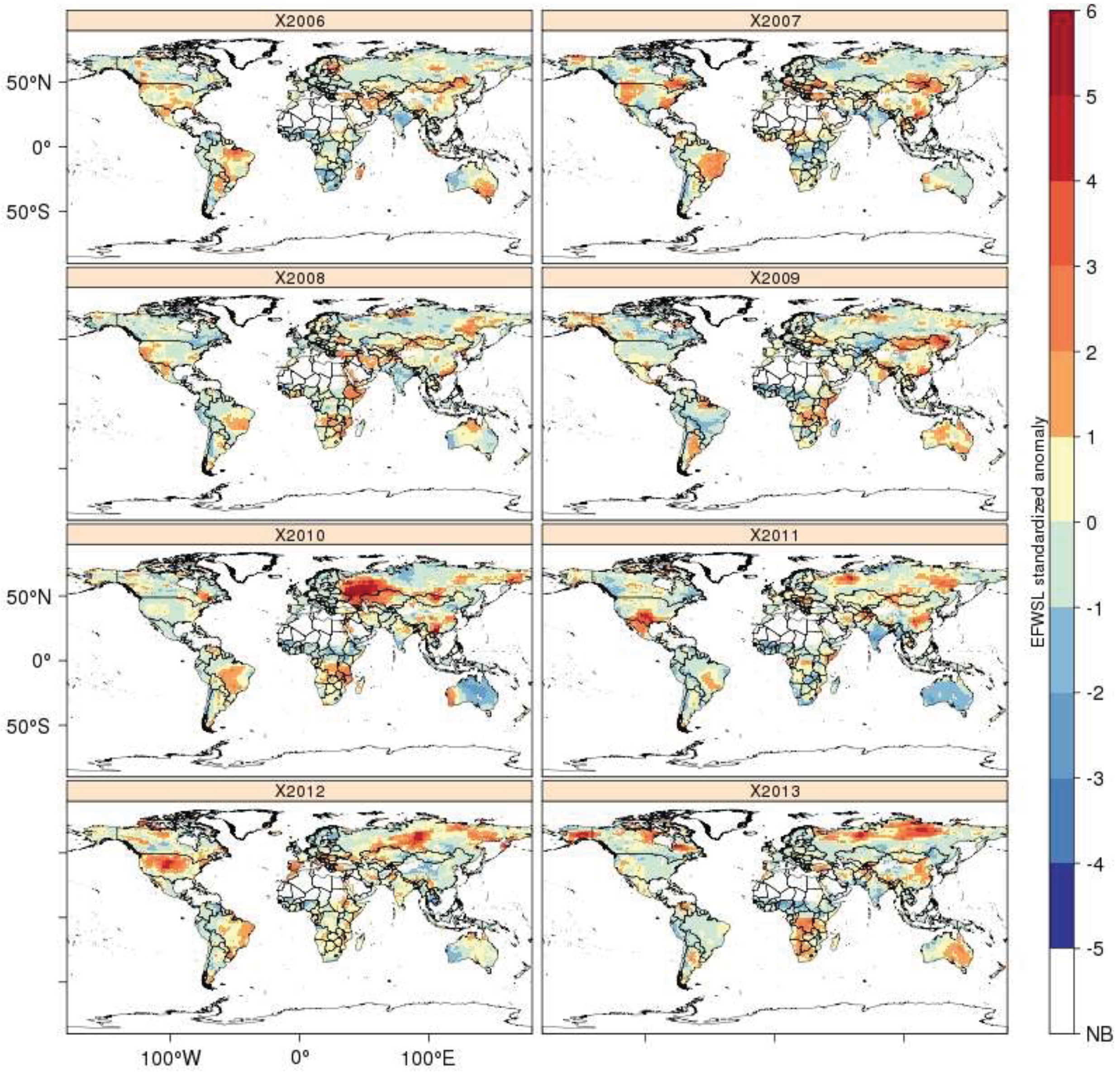

Supplementary Figure 4 – Maps of standardized anomalies of Fire Weather Season Length (Equation 5) from 2006-2013.

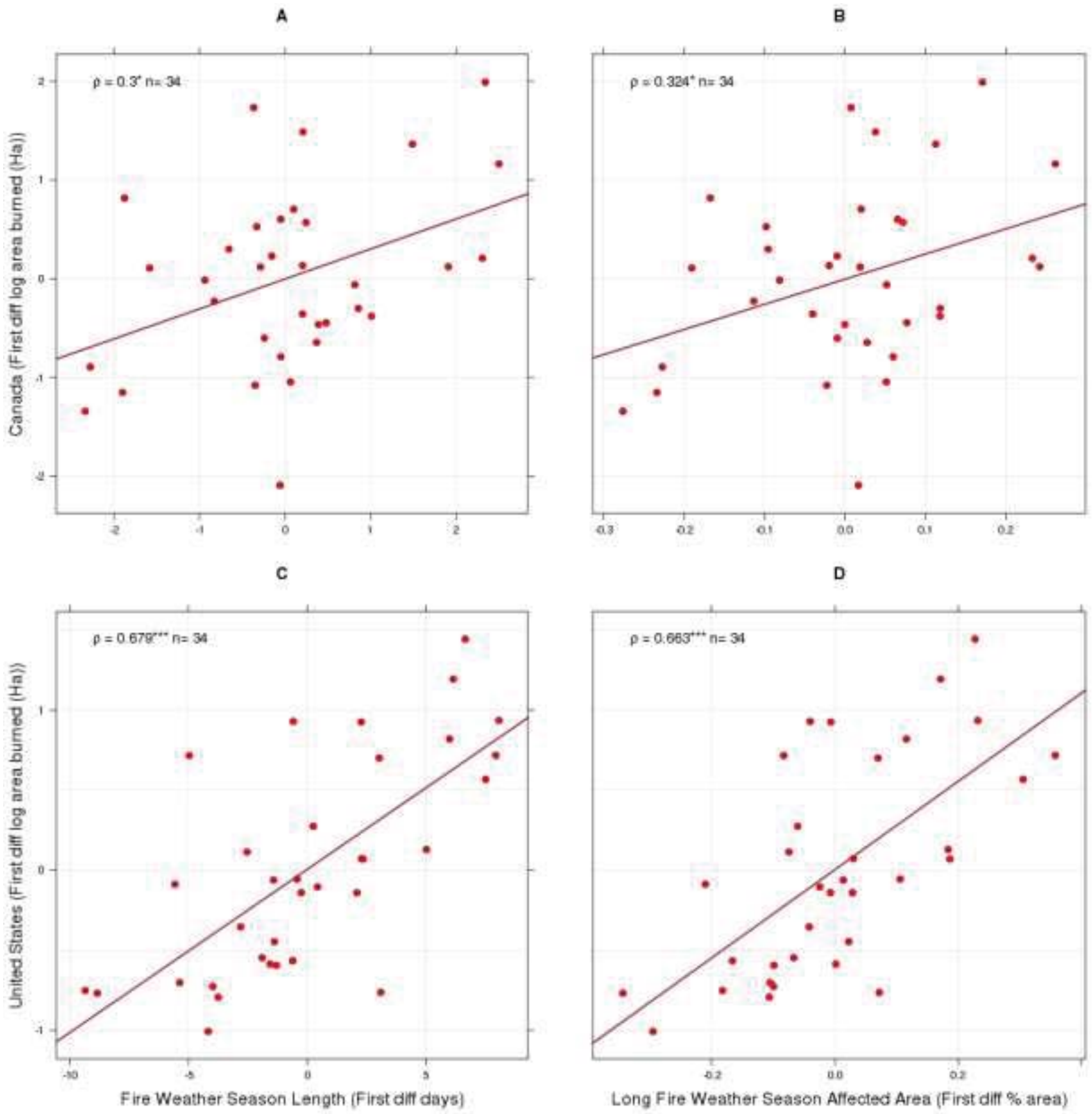

Supplementary Figure 5 – Comparisons of first-difference detrended spatial mean fire weather season length and long fire weather season affected area across Canada (A,B) and the United States (C,D) from 1979-2013. Neither fire weather season length nor long fire weather season affected was significantly related to burned area in Canadian forests but both season metrics were significantly related to burned area across the United States. Red lines show the estimated Theil-Sen trend slopes. Values reported in plot upper left are Spearman's rank-order correlation and corresponding significance (* p < 0.01, ** p < 0.05, * p < 0.1, NS Not Significant)**

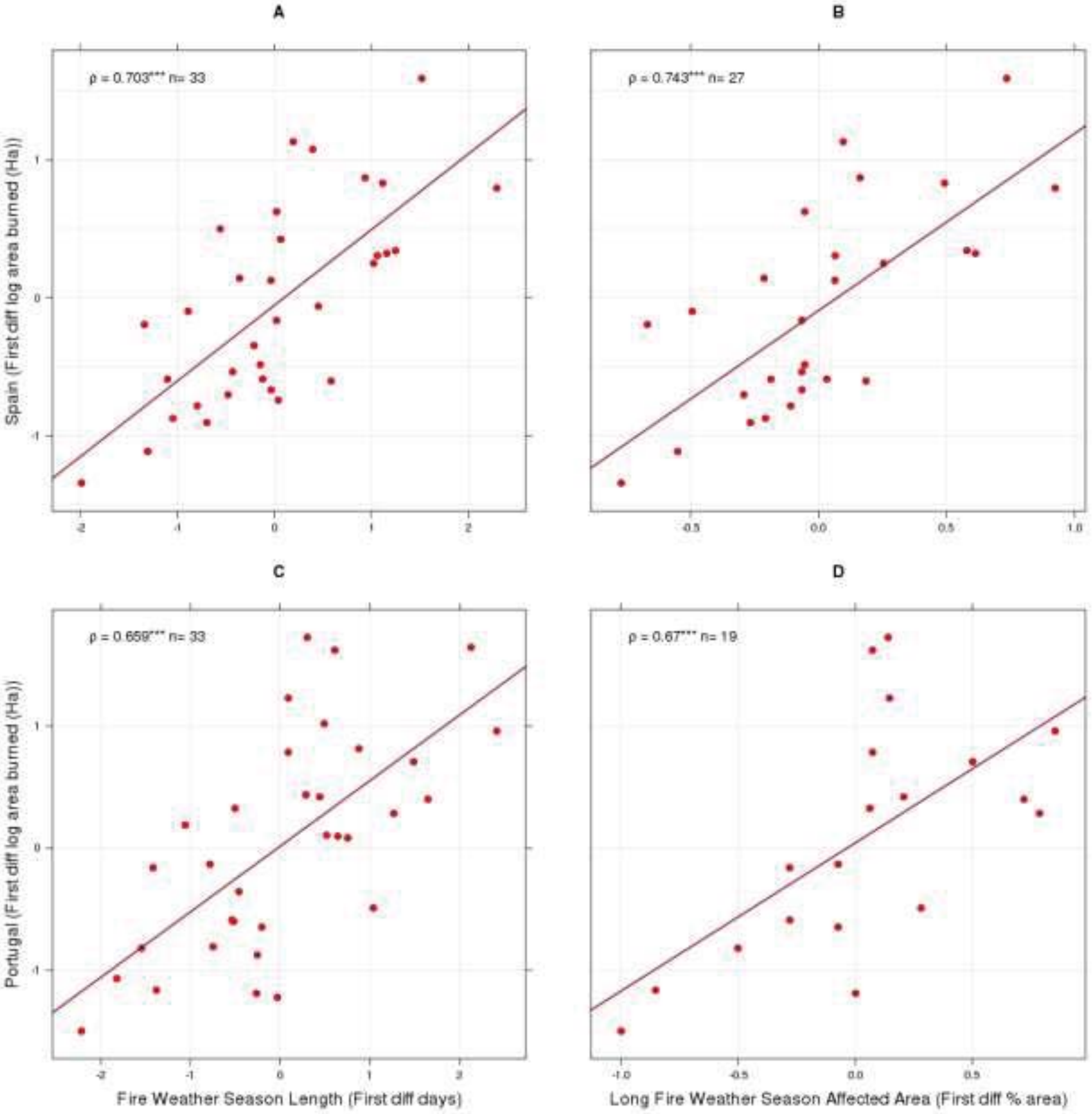

Supplementary Figure 6 – Comparisons of first-difference detrended spatial mean fire weather season length and long fire weather season affected area across Spain (A,B) and the Portugal (C,D) from 1980-2013. Both season metrics were significantly related to burned area across Spain and Portugal. Years where long fire weather season affected area was zero were excluded from analysis; therefore sample sizes for panels B and D are variable. Red lines show the estimated Theil-Sen trend slopes. Values reported in plot upper left are Spearman's rank-order correlation and corresponding significance (* p < 0.01, ** p < 0.05, * p < 0.1, NS Not Significant).**

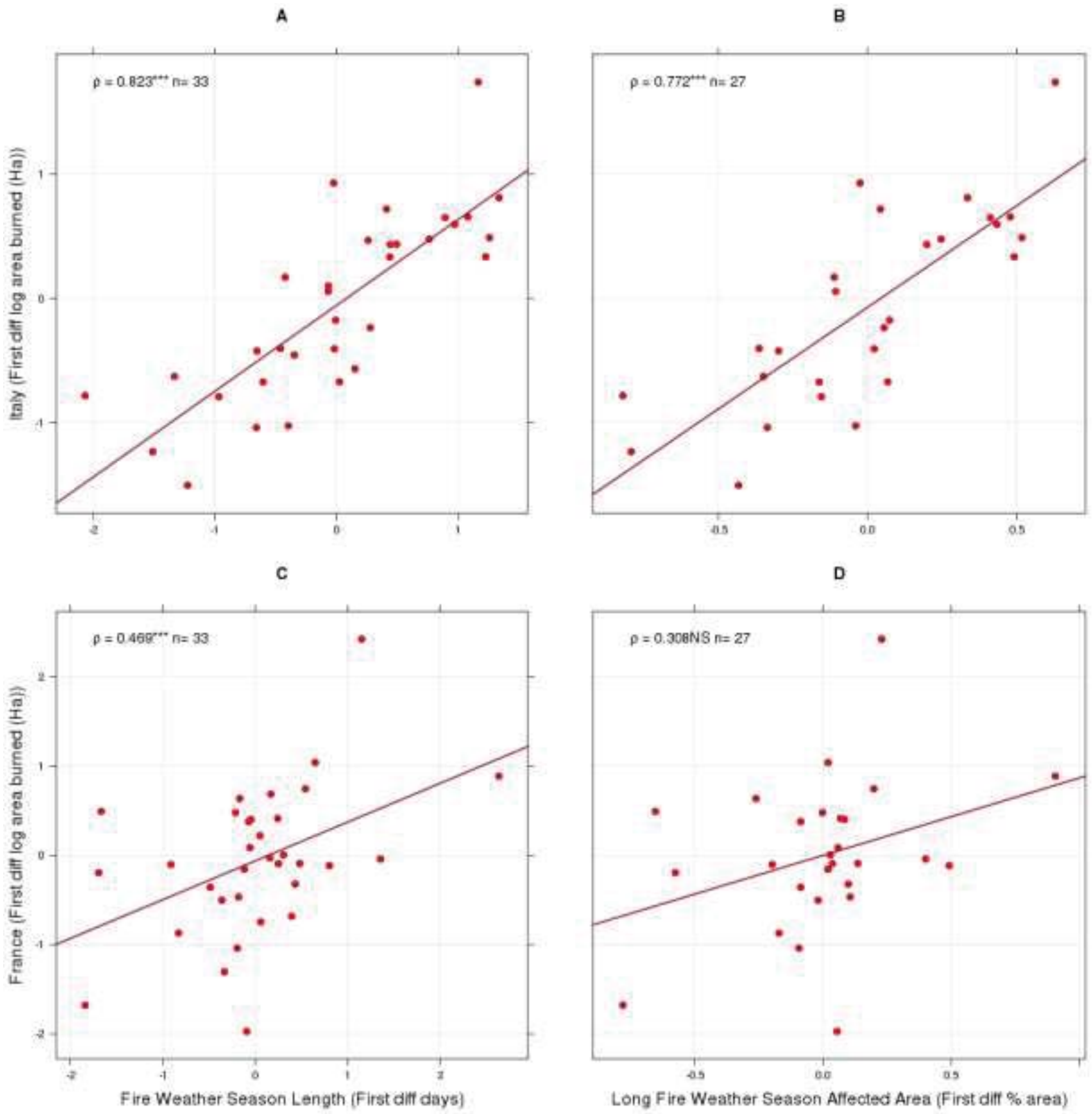

Supplementary Figure 7 – Comparisons of first-difference detrended spatial mean fire weather season length and long fire weather season affected area across Italy (A,B) and France (C,D) from 1980-2013. Fire weather season length was significantly related to burned area across both Italy and France and long fire weather season affected area was significantly correlated to burned area in Italy. Years where long fire weather season affected area was zero were excluded from analysis; therefore sample sizes for panels B and D are variable. Red lines show the estimated Theil-Sen trend slopes. Values reported in plot upper left are Spearman's rank-order correlation and corresponding significance (* p < 0.01, ** p < 0.05, * p < 0.1, NS Not Significant).**

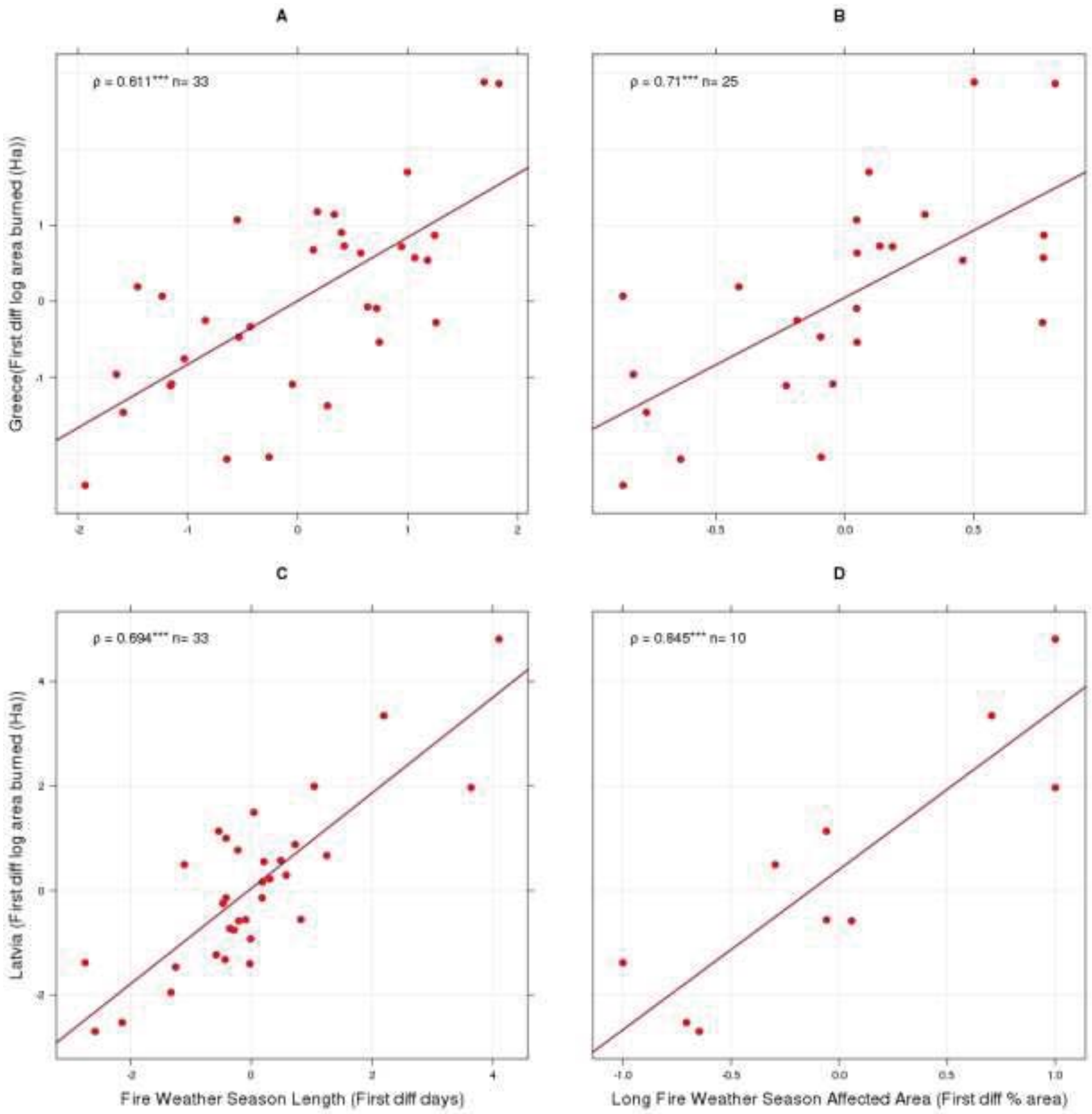

Supplementary Figure 8 – Comparisons of first-difference detrended spatial mean fire weather season length and long fire weather season affected area across Greece (A,B) and Latvia (C,D) from 1980-2013. Fire weather season length was significantly related to burned area across both Greece and Latvia and long fire weather season affected area was significantly correlated to burned area in Latvia. Years where long fire weather season affected area was zero were excluded from analysis; therefore sample sizes for panels B and D are variable. Red lines show the estimated Theil-Sen trend slopes. Values reported in plot upper left are Spearman's rank-order correlation and corresponding significance (* p < 0.01, ** p < 0.05, * p < 0.1, NS Not Significant).**

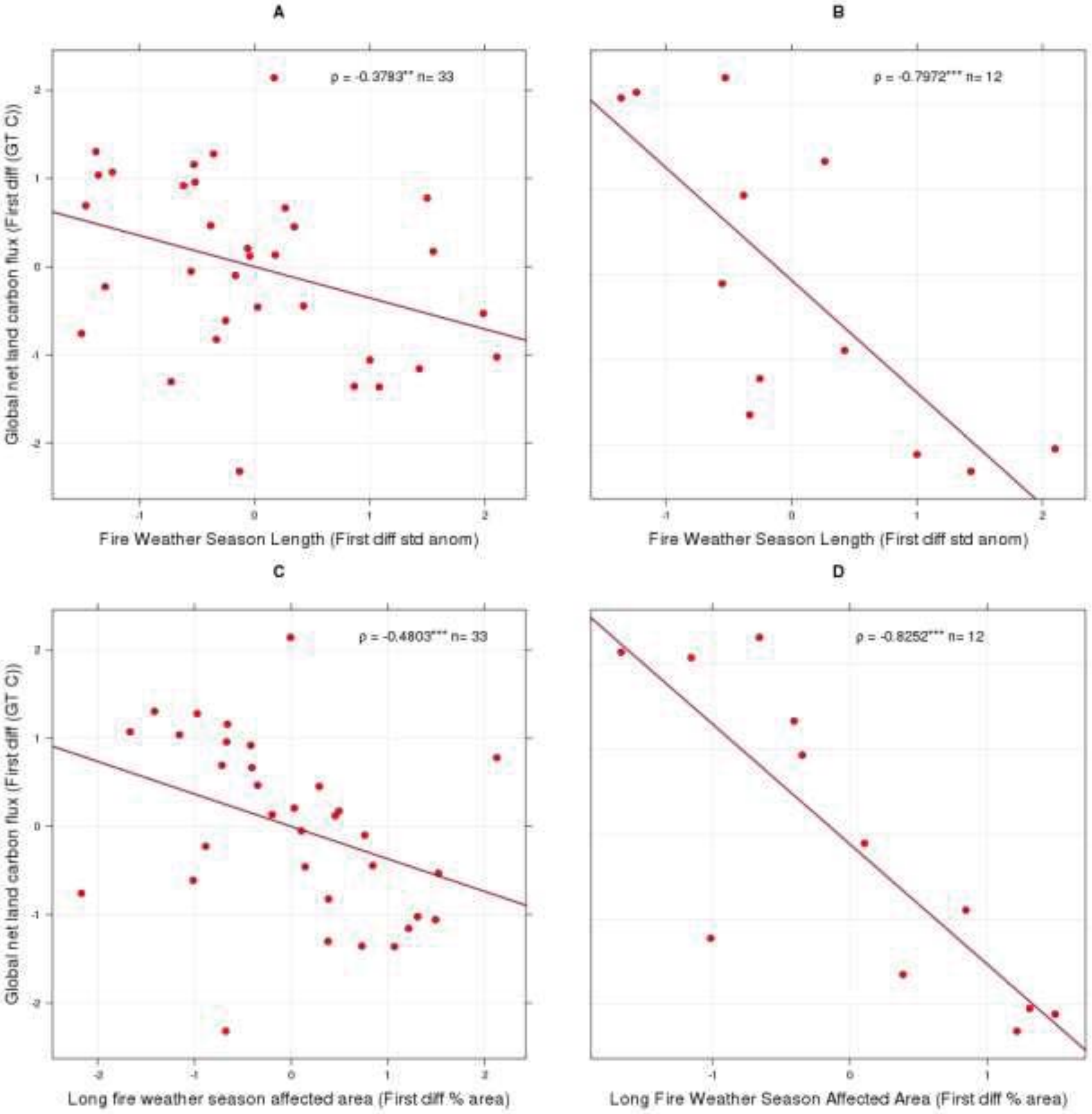

Supplementary Figure 9 – Comparisons of global fire weather season length and long fire weather season affected area to global net land carbon flux from 1979-2012 (A,C respectively) and for the most recent time period where MODIS burned area data were available (2001-2012) (B,D). Values reported in plot upper right are Spearman's rank-order correlation, corresponding significance (* $p < 0.01$, ** $p < 0.05$, * $p < 0.1$, NS Not Significant) and sample size.**

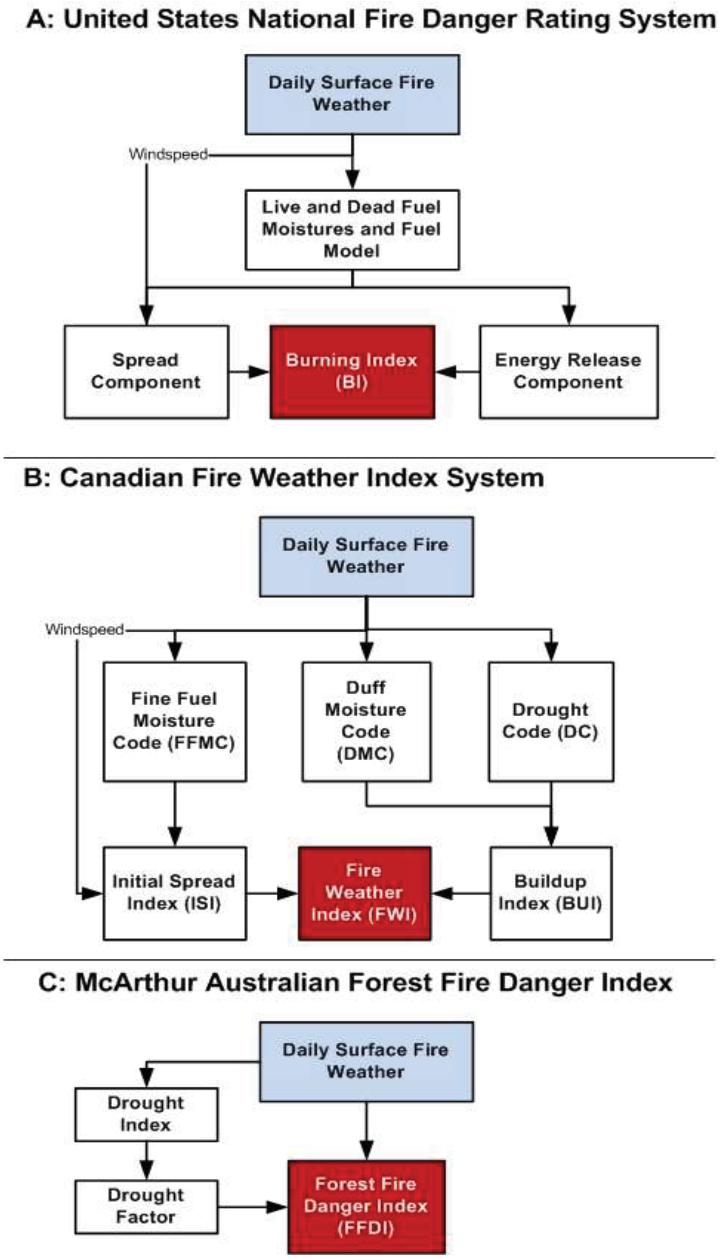

Supplementary Figure 10 – Simplified flow diagram of the US Burning Index (BI), the Canadian Fire Weather Index (FWI) and the Australian Forest Fire Danger Index (FFDI).

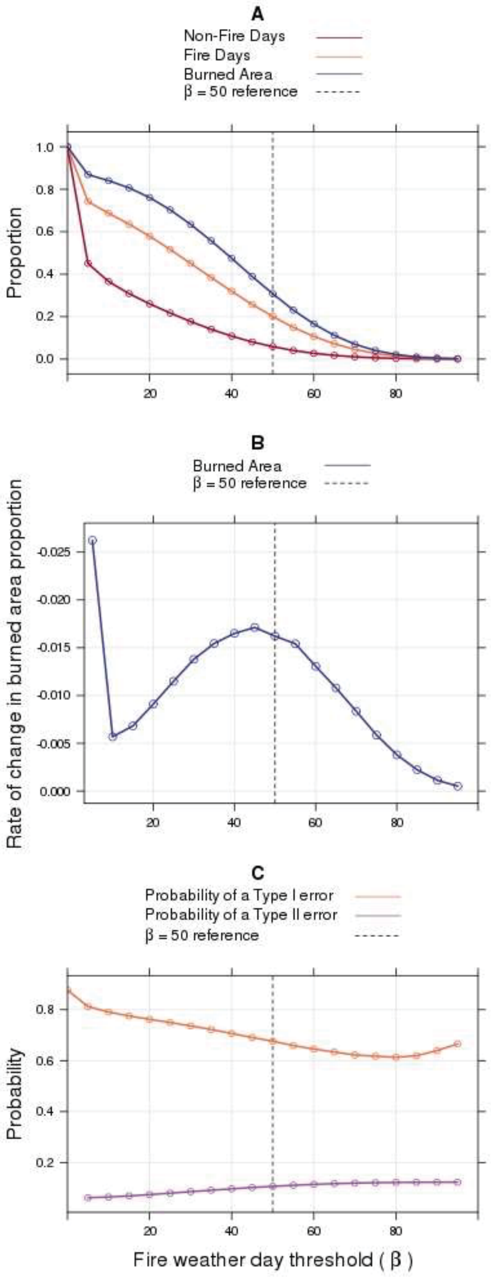

Supplementary Figure 11 – Effect of the selection of β (Equation 2) on the ability of the fire weather day index (FWDI) to capture global fire activity recorded between 2001 and 2012 in the most recent version of the Global Fire Emissions Database (GFED4). The reference value (β=50) was selected *a priori* from literature but these figures show that it represents the best balance between capturing a large portion of the burned area while minimizing the chance of mis-classifying fire days. When β=50, our Fire Weather Day Index captures 20.1% of fire days and 30.8% of the total burned area. Although reducing β captures more fire days and more burned area (A), it also captures an increasing proportion of non-fire days yielding an Ensemble Fire Weather Season Length (EFWSL) that is more representative of days without fire activity. The slope of the curve relating the proportion of burned area to β is steepest near β =50 indicating that reducing the value of β much below 50 captures incrementally smaller gains in total burned area (B). Additionally, reducing β also increases the likelihood making a Type I error (C), where a Fire Weather Day is predicted but no burned area is observed.

Supplementary Tables:

Supplementary Table 1 – Trends in global annual summary meteorological variables and their respective affected area.

Variable	Mean annual value trend		Affected area trend (change in area / yr)	
	**Slope**	**p-value**	**Slope**	**p-value**
Mean annual maximum temperature (°C)	0.0184°C yr^{-1}	<0.0001	0.628	<0.0001
Mean annual minimum relative humidity (%)	-0.0127% yr^{-1}	0.0356	NS	NS
Mean annual total precipitation (mm)	NS	NS	NS	NS
Mean annual total rain-free period (days)	0.131 days yr^{-1}	0.000985	0.162	0.00162
Mean annual maximum windspeed (km hr^{-1})	0.000691km hr^{-1} yr^{-1}	0.0332	NS	NS

Non-parametric tests examining temporal trends in ensemble-mean global fire weather variables and the area affected by unusual events for mean annual maximum temperature, mean annual minimum relative humidity, total annual precipitation, mean annual rain-free days and maximum 10m windspeed. (NS Not Significant). Slopes were estimated using the Theil-Sen non-parametric trend slope estimator and significance tests were performed using the Mann-Kendall trend test following a four-step approach to reduce the effects of serial autocorrelation on significance tests[1].

Supplementary Table 2 – NCEP Reanalysis and DOE Reanalysis II data variables used to derive fire danger indices.

Variable	Level	Units	Derived Quantity
Maximum temperature	2 m	K	Maximum
Minimum temperature	2 m	K	Minimum
Pressure	Surface	Pa	Mean
Specific Humidity	2 m	kg/kg	Mean
Precipitation Rate	Surface	kg/m2/s	Duration and Total Amount
U/*V* wind	10 m	m/s	Maximum windspeed
Water equivalent of actual snow depth	Surface	kg/m2	Snow flag

Underlying variables used to calculate fire danger indices from both the NCEP Reanalysis and the Reanalysis II datasets. All variables were derived from 4 times daily grids at 192X94 (~2° resolution) Gaussian grid resolution.

Supplementary Table 3 – ECMWF ERA Interim Reanalysis data variables used to derive fire danger indices.

Variable	Level	Units	Summary
Dewpoint temperature	2 m	K	Mean
Air temperature	2 m	K	Maximum / Minimum
Total precipitation	Surface	m	Duration and Total Amount
Snow depth	Surface	m	Snow flag
U/V wind	10 m	m/s	Maximum

Underlying variables used to calculate fire danger indices from the ECMWF Interim Reanalysis. All variables were derived from 8 times daily grids at 479x239 (~0.75° resolution) geographic grid.

Supplementary Table 4 – Summary of classification accuracy of daily MODIS burned area based on daily Fire Weather Day Indices.

Beta	Type I Error (False Positive)	Correct Decision (True Positive)	Correct Decision (True Negative)	Type II Error (False Negative)
	Conditional Probability of **No** MODIS BA detection given a Fire Weather Day	Conditional Probability of a MODIS BA detection given a Fire Weather Day	Conditional Probability of **No** MODIS BA detection given a Non-Fire Weather Day	Conditional Probability of a MODIS BA detection given a Non-Fire Weather Day
**β**	**Pr(BA=0|FWDI=1)**	**Pr(BA>0|FWDI=1)**	**Pr(BA=0|FWDI=0)**	**Pr(BA>0|FWDI=0)**
0	0.88	0.12	Undefined	Undefined
5	0.81	0.19	0.94	0.06
10	0.79	0.21	0.94	0.06
15	0.78	0.22	0.93	0.07
20	0.76	0.24	0.93	0.07
25	0.75	0.25	0.92	0.08
30	0.74	0.26	0.91	0.09
35	0.72	0.28	0.91	0.09
40	0.71	0.29	0.90	0.10
45	0.69	0.31	0.90	0.10
50	0.68	0.32	0.89	0.11
55	0.66	0.34	0.89	0.11
60	0.65	0.35	0.89	0.11
65	0.63	0.37	0.88	0.12
70	0.62	0.38	0.88	0.12
75	0.62	0.38	0.88	0.12
80	0.61	0.39	0.88	0.12
85	0.62	0.38	0.88	0.12
90	0.64	0.36	0.88	0.12
95	0.67	0.33	0.88	0.12

Contingency table values of the ability of the Fire Weather Day Index to accurately classify the probability of a MODIS burned area day.

Supplementary Methods:

Wildfire Danger Indices

Global reanalysis data, summarized to daily values , were input into three common wildland fire danger rating systems: The United States National Fire Danger Rating System (USNFDRS), the Canadian Fire Weather Index System (CFWIS) and the McArthur Forest Fire Danger Index (FFDI).

A single C++ based library was created that allows the simultaneous calculation all fire danger indices. The USNFDRS library is the same library used operationally to assess fire danger for all fire management applications. All USNFDRS fuel moistures, components and indices were computed following Bradshaw et al.[2]. To ensure that indices were comparable spatially and temporally and to allow the combination of US indices with other indices, we constrained all calculations to Fuel Model G as recommended by Andrews[3]. This fuel model heavily weights long time-lag fuels, such as 100 hour and 1000 hour classes, and thus best represents seasonal wetting and drying cycles[3]. The USNFDRS culminates in a single index, called the Burning Index (BI), that is related to both the expected rate of spread and heat release of an initiating fire. The US Burning Index is calculated by combining meteorological variables to first calculate time-lag fuel moisture contents. Fuel moisture contents are then combined to calculate the Spread Component and the Energy Release Component which were then used to calculate the Burning Index (Supplementary Figure 10A).

The Canadian Forest Fire Weather Index (FWI) was computed following Van Wagner and Pickett[4], with variable latitude adjustment factors to allow these indices to be calculated globally[5]. Similar to the US system, Canadian FWI are aligned as a two-step process. First, three “Codes” are calculated using surface weather data and then these codes are combined into “Indices”. Surface weather was used to calculate the Fine Fuel Moisture Code, the Duff Moisture Code and the Drought Code and these Codes were combined to calculate the Initial Spread Index (ISI) and the Build-up Index (BUI). Finally, the ISI and BUI are combined to calculate the Fire Weather Index (Supplementary Figure 10B). Generally, both the US and Canadian systems are applied using afternoon weather (either 1200 or 1300 local standard time) but to ensure global applicability, all calculations were performed substituting daily maximum temperature and minimum relative humidity for midday weather. Further, in areas with ephemeral snow cover, both the US and Canadian indices were initialized to start-up values each season anytime snow cover was present. For consistency between indices, the

effects of prolonged drought, typically accounted for by ‘overwintering’ the Drought Code, were ignored[6].

The McArthur (Australian) Forest Fire Danger Index (FFDI) was calculated following the logic presented by McArthur and expressed as equations by Noble[7]. The Drought Factor for these equations was calculated using the improved formula presented by Griffiths[8] driven by the Keetch-Byram Drought Index (Supplementary Figure 10C). KBDI was calculated using daily maximum temperature and precipitation from each reanalysis dataset and mean annual precipitation values from the WorldClim climate dataset[9]. The BI, FWI, and FFDI were each calculated using the NCEP Reanalysis, the NCEP DOE Reanalysis II and the ECWMF Interim Reanalysis data to yield nine independent daily fire danger indices at 0.75°×0.75° spatial resolution for the ECWMF and an approximately 2° × 2° spatial resolution for the NCEP and NCEP DOE Reanalysis II datasets.

Developing metrics of wildfire weather season length

While some systems, such as the Canadian Fire Weather Index System, have established thresholds for its indices that relate to periods where fires spread actively[10, 11], the other two systems have no similar thresholds. Therefore, a common way of normalizing and comparing all three indices across space and time is needed. The magnitudes of each of the three BI, FWI, and FFDI indices vary spatially and temporally, by up to two orders of magnitude, making it necessary to normalize each daily index relative to its historical range for that location. An established practice is to normalize each daily value based on the historical maximum and minimum recorded in the grid cell[12]. This procedure ensures that time-series of fire danger indices can be compared between re-analysis projects and between grid cells across the globe. Daily fire danger indices were normalized in each grid cell as follows:

$$FDI_{Norm_{ij}} = \left[\frac{FDI_{ij} - FDI_{Min}}{FDI_{Max} - FDI_{Min}}\right] * 100 \qquad \text{Equation 1}$$

Where FDI_{ij} is the daily fire danger index for a given location for day i of year j, FDI_{Min} and FDI_{Max} are the historical daily minimum and maximum fire danger indices for that location for the entire time series, and FDI_{Normij} is the normalized, daily fire danger index. FDI_{Normij} is bounded by 0 and 100 indicating the historical minimum and maximum fire danger index for each grid cell, respectively.

Fire Weather Days

Once normalized, we expressed each day as either a fire weather day or a non-fire weather day as follows:

$$FWDI_{ij} = \begin{cases} 1: FDI_{Norm_{ij}} \geq \beta \\ 0: FDI_{Norm_{ij}} < \beta \end{cases} \quad \text{Equation 2}$$

Where FDI_{Normij} is the daily, normalized fire danger index calculated from Equation 1 and $FWDI_{ij}$ is a fire weather day index indicating that wildfire potential exceeded the threshold β on a particular day. Setting the threshold of β = 0 implies that all days are fire weather days, and increasing the value of β corresponds to more conducive fire weather conditions. For our analysis, we set β=50 *a priori* based on the established technique commonly used to identify phenological events such as leaf out and leaf senescence using satellite time series of vegetation indices[13]. A universal threshold of β=50 lies at the midpoint between the lower and upper values recorded over the entire time-series in each grid cell, and as such, standardizes the comparison of fire weather across space and time.

We evaluated our *a priori* choice of β to assess the ability of the fire weather day index to capture daily fire activity by associating the normalized daily fire danger index (FDI_{Normij}) with the daily burned area recorded in the most recent version of Global Fire Emissions Database (GFED4)[14]. GFED4 summarizes the daily 500m Collection 5.1 MODIS direct broadcast (DB) burned area product (MCD64A1) at 0.25° spatial resolution (since August 2000)[15]. We used 11 years of daily

data (2001 to 2012) resampled to the gridded domain of FDI_{Norm}. Synchronizing fire danger and fire activity in each grid cell yielded 9 daily time series of FDI_{Norm} (one for each fire danger index and reanalysis data combination) and a daily time series of burned area. We varied the β threshold in 1 Equation 2 that is used to determine the Fire Weather Day Index and we quantified the mean proportion of fire days (i.e., a day with at least one 500m burned area pixel in a grid cell), mean proportion of non-fire days and the mean proportion of burned area captured by the 9 values of the 4 FWDI (Supplementary Figure 11A).

Results indicate that our *a priori* selection of β=50 captures 20.1% of fire days and 30.8% of the total global burned area. Additionally, it aligns closely with the point of maximum rate of change of captured burned (Supplementary Figure 11A), thus the FWDI captures smaller gains in burned area as the threshold β varies both above and below β=~45 (Supplementary Figure 11B). Although reducing β necessarily captures more fire days and more burned area (Supplementary Figure 11A), selecting a lower β threshold leads to a higher probability of making a Type I error, where we classify a fire weather day but MODIS does not detect burned area on that day (Supplementary Figure 11C and Supplementary Table 4). Conversely, increasing β above 50 to 80 minimizes the Type I errors (Supplementary Table 5) but it only captures 1.4% of the global fire days and 2.2% of the burned area (Supplementary Figure 11A). Despite being selected *a priori*, our choice of β=50 is the best compromise between minimizing the probability of a Type I error while maximizing the burned area captured by our Fire Weather Day Index. Finally, our index is based only on fire weather; it does not incorporate any information about available fuels or sources of ignition. While we expect that if ignited, a fire will spread more rapidly on a fire weather day, we do not expect to explain all the variations in global burned area with weather alone, especially given that human-ignited fires burning outside the seasonal peak of fire weather account for a large proportion of the annual global burned area[16].

Ensemble Fire Weather Season Length

Whilst the same logic is commonly used to determine growing season length from satellite-derived vegetation indices[13, 17], our goal is to estimate a fire weather season length. We calculate an annual "Fire Weather Season Length" that represents the number of days (not necessarily continuous) during each calendar year at a given location that observed high fire danger and thus experienced weather conditions most conducive to ignition and burning, as follows:

$$\boldsymbol{FWSL_j = \sum_{i=1}^{365|366} FWDI_{ij}} \qquad \text{Equation 3}$$

Where $FWDI_{ij}$ is the daily fire weather day index calculated from Equation 2 and $FWSL_j$ is the annual Fire Weather Season Length (FWSL) in days for year *j*. FWSL is calculated in each grid cell for each fire danger index (BI, FWI, FFDI) and reanalysis dataset (NCEP, NCEP II and ECMWF Interim), yielding nine annual fire weather season length values for each grid cell. Because climate studies using multi-model ensembles are generally superior to single model approaches[18], an ensemble-mean fire weather season length is calculated as follows:

$$\boldsymbol{EFWSL_j = \frac{\sum_{n=1}^{9} FWSL_{jn}}{9}} \qquad \text{Equation 4}$$

Where $FWSL_j$ is the FWSL calculated for a given location and a given index / model combination (*n*) and $EFWSL_j$ is the Ensemble-mean Fire Weather Season Length across all nine index / model combinations in a grid cell for year *j*.

To allow combination of disparate raster resolutions into a single ensemble dataset, the coarse fire weather season length raster datasets derived from BI, FWI and FFDI using the NCEP and NCEP II datasets were resampled to match the 0.75° x 0.75° resolution of the ECMWF Interim Reanalysis dataset using the nearest neighbor method. After resampling, all nine fire weather season length

raster datasets were averaged for each year from 1979 to 2013 to produce an Ensemble Fire Weather Season Length raster dataset at 0.75° x 0.75° resolution (Equation 4).

We also calculated a standardized anomaly by expressing the annual $EFWSL_j$ in each grid cell relative to its 35-year mean and standard deviation as follows:

$$\boldsymbol{EFWSL_{Anom_j}} = \frac{\boldsymbol{EFWSL_j} - \overline{\boldsymbol{EFWSL}}}{\boldsymbol{\sigma EFWSL}} \qquad \textbf{Equation 5}$$

Where $EFWSL_j$ is the annual ensemble mean fire weather season length calculated above, $\overline{EFWSL}$ and $\sigma EFWSL$ are the 35 year mean and standard deviation of EFWSL for that location and $EFWSL_{Anomj}$ is the standardized anomaly of EFWSL for year j for a given location. All analyses are performed on either the ensemble fire weather season length or its anomalies. For simplicity, the term "Fire Weather Season Length" is used throughout to denote the Ensemble Fire Weather Season Length.

Analysis

We used a simple mask that delineated vegetated and non-vegetated areas that was developed approximately in the middle of the study period[19]. Pixels were considered vegetated if they were not listed as: water, bare ground or urban and built-up in the land cover dataset. All spatial mean trends were calculated using a cell-area-weighted average because the cell sizes of the geographic projection that we used for the dataset vary from the equator to the pole. Trend significance was evaluated using the Mann-Kendall trend tests[20] by following a four-step trend-free prewhitening (MKTFPW) approach that reduces the impact of serial autocorrelation on significance tests[1]. Trend slopes were estimated using the non-parametric Theil-Sen (TS) trend estimator[21, 22]. Further, pixel-based trend analyses were performed using the MKTFPW and the TS trend slope estimator and all pixels with significant fire weather season length trends ($p < 0.05$) were mapped to produce Figure 3A. Because climatic changes can lead to either a persistent trend or an increase in rare events, we

also examined the changes in the global frequency of long fire weather seasons. A long fire weather season was defined as any year where a given pixel's fire weather season length was more than 1 standard deviation above the mean. We then summed the total land area of all vegetated pixels that experienced these unusually long fire weather seasons each year. Hereafter, this metric is referred to as: long fire weather season affected area. We plotted these global affected area sums over time from 1979 to 2013 (Figure 2B). Finally, we developed a simple way to spatially depict areas that have witnessed changes in the frequency of these long fire seasons. For each pixel, we determined the number of times that fire weather season length standard anomalies exceeded one standard deviation from the mean. Hereafter, any year with an anomalous fire season length will be termed an event year. We then summed the number of events from 1979-1996 (18 years) and we summed the number of events from 1996-2013 (18 years), where 1996 overlaps to yield the same number of years in each period. We mapped the difference between the number of events in the second period and the number of events in the first period and expressed that difference as a percentage change in the number of years (Figure 3B).

Comparison to country-wide reported burned area

To determine the degree to which the fire weather season length is related to observed fire activity, we compared yearly spatial-mean fire weather season length values to published total annual area burned on US federal lands, documented by the National Interagency Fire Center (NIFC)[23], area burned across Canadian Forests[24] and for six European countries (Spain, Portugal, France, Italy, Greece and Latvia), where annual burned area reports were available from 1980-2013. Two comparisons were made for the United States. We first compared mean fire weather season length across the United States (including Alaska) to annual burned area from 1992-2013. Fire occurrence data prior to 1992 are often incomplete and thus reported burned area may or may not accurately reflect actual burned area[25]. However, because burned area estimates are published by the National

Interagency Fire Center for our entire study period, we also compared mean fire weather season length to burned area for the full period, with the caveat that data prior to 1992 may be somewhat incomplete. We compared mean annual fire weather season length and long fire weather season affected area for each country to their reported burned area. All time series were first-difference detrended to ensure stationarity and all comparisons were performed using the Spearman's rank-order correlation. Correlations are reported in Table 4 and plots of all comparisons are shown in (Supplementary Figure 5-8).

Regional fire weather season length trends

Global patterns in fire weather season length were further constrained to examine changes across continents and biomes. A coarse-level biome map was derived from the World Wildlife Fund (WWF) Ecoregions biome classification[26]. Since the spatial resolution of the climate datasets was coarser than some of the biome delineations, we aggregated multiple WWF biomes into eight broad biome classes (See Supplementary Table 5). The same methods used to assess the global trends (defined above) were also used to assess (i) continental, (ii) biome, and (iii) continental × biome trends. Continental boundaries were used to develop an analysis map, excluding Antarctica and combining Europe and Asia, as well as Australia and New Zealand. The continental and biome analysis maps were used to assess changes in continental average (Table 1), global biome average (Table 2), and continent × biome average fire weather season length as well as long fire weather season affected area (Table 3). Finally, five number summaries (minimum, first quartile, median, third quartile and maximum) were generated for all pixels that fall within each continent x biome combination that had significant trends in fire weather season length to assess central tendencies and range of fire weather season length changes (Table 6).

Comparisons to global carbon fluxes

To assess the degree to which fire weather season lengths are potentially coupled to global carbon emissions, we compared the mean global fire weather season length and the long fire weather season

affected area to the global annual net land carbon uptake computed from the Global Carbon Budget dataset from 1979-2012[27]. This carbon budget decomposes global annual carbon fluxes and a global annual carbon 'land sink' is computed as follows:

$$LndSnk = FF\text{-}CPE + LUCE - AtmGr - OcnSnk \qquad \text{Equation 6}$$

Where *LndSnk* is the land carbon sink, *FF-CPE* are fossil fuel and cement production emissions[28], *LUCE* is land-use change emissions[29], *AtmGr* is the atmospheric CO_2 growth rate and *OcnSnk* is the ocean carbon sink[27]. All global fluxes are reported in billions of tons of carbon per year (GtC per yr). Because we were interested in comparing our global fire weather season length metric to variations in global land carbon flux variations, which includes components of the global land sink as well as components of land-use change emissions, we computed a global net land carbon flux as follows:

$$NetLndCFlux = LndSnk - LUCE \qquad \text{Equation 7}$$

Where *NetLndCFlux* is the global net land carbon flux and *LndSnk* and *LUCE* are explained above. This metric is positive when there is a net carbon uptake by the land surface and negative when there is a net loss of terrestrial carbon. It accounts for potential variations in land-use change emissions as well as other potential wildfire-derived carbon emissions that are not a direct result of land-use change activities but that are embedded in the total global land carbon flux. Two comparisons were performed: one for the entire available, overlapping time series (1979-2012), and another where correlations were constrained to the time period that satellite burned area observations were available from the Moderate Resolution Imaging Spectroradiometer (MODIS) (2001-2012), and thus when estimates of land-use change carbon emissions were more certain[14]. All time series were first-difference detrended to ensure stationarity and all comparisons

were performed using the Spearman's rank-order correlation. Correlations were also examined between continent x biome-mean fire weather season length, long fire season affected area and global net land carbon flux to assess where fire might be most coupled to the global carbon cycle.

Supplementary References:

1. Yue S, Pilon P, Phinney B, Cavadias G. The influence of autocorrelation on the ability to detect trend in hydrological series. *Hydrological Processes* **16**, 1807-1829 (2002).

1. Yue S, Pilon P, Phinney B, Cavadias G. The influence of autocorrelation on the ability to detect trend in hydrological series. *Hydrological Processes* **16**, 1807-1829 (2002).

2. Bradshaw LS, Deeming JE, Burgan RE, Cohen JD. The 1978 NFDRS: technical documentation. *USDA Forest Service, Gen Tech Rep INT-169*, (1983).

3. Andrews PL, Loftsgaarden DO, Bradshaw LS. Evaluation of fire danger rating indexes using logistic regression and percentile analysis. *International Journal of Wildland Fire* **12**, 213-226 (2003).

4. Van Wagner CE, Pickett TL. *Equations and FORTRAN program for the Canadian forest fire weather index system*. Canadian Forestry Service (1985).

5. Lawson BD, Armitage OB. *Weather guide for the Canadian forest fire danger rating system*. Northern Forestry Centre (2008).

6. Turner JA, Lawson BD. Weather in the Canadian forest fire danger rating system. A user guide to national standards and practices. *Report Pacific Forest Research Centre*, (1978).

7. Noble IR, Gill AM, Bary GAV. McArthur's fire-danger meters expressed as equations. *Australian Journal of Ecology* **5**, 201-203 (1980).

8. Griffiths D. Improved Formula for the Drought Factor in McArthur's Forest Fire Danger Meter. *Australian forestry* **62**, 202-206 (1999).

9. Hijmans RJ, Cameron SE, Parra JL, Jones PG, Jarvis A. Very high resolution interpolated climate surfaces for global land areas. *International Journal of Climatology* **25**, 1965-1978 (2005).

10. Podur J, Wotton BM. Defining fire spread event days for fire-growth modelling. *International Journal of Wildland Fire* **20**, 497-507 (2011).

11. Wang X, *et al.* The potential and realized spread of wildfires across Canada. *Global Change Biology*, (2014).

12. Viegas DX, Bovio G, Ferreira A, Nosenzo A, Sol B. Comparative study of various methods of fire danger evaluation in southern Europe. *International Journal of Wildland Fire* **9**, 235-246 (1999).

13. White MA, Thornton PE, Running SW. A continental phenology model for monitoring vegetation responses to interannual climatic variability. *Global Biogeochemical Cycles* **11**, 217-234 (1997).

14. Giglio L, Randerson JT, van der Werf GR. Analysis of daily, monthly, and annual burned area using the fourth generation global fire emissions database (GFED4). *Journal of Geophysical Research: Biogeosciences* **118**, 317-328 (2013).

15. Giglio L, Loboda T, Roy DP, Quayle B, Justice CO. An active-fire based burned area mapping algorithm for the MODIS sensor. *Remote Sensing of Environment* **113**, 408-420 (2009).

16. Le Page Y, Oom D, Silva JMN, Josson P, Pereira JMC. *Global Ecology and Biogeography* **19**, 575-588 (Seasonality of vegetation fires as modified by human action: observing the deviation from eco-climatic fire regimes).

17. de Beurs KM, Henebry GM. Spatio-temporal statistical methods for modelling land surface phenology. In: *Phenological research* (ed^(eds). Springer (2010).

18. Hagedorn R, Doblas-Reyes FJ, Palmer TN. The rationale behind the success of multiâ€• model ensembles in seasonal forecasting--I. Basic concept. *Tellus A* **57**, 219-233 (2005).

19. Hansen MR, DeFries RS, Townshend JRG, Sohlberg R. Global land cover classification at 1km resolution using a decision tree classifier. *International Journal of Remote Sensing* **21**, 1331-1365 (2000).

20. Mann HB. Nonparametric tests against trend. *Econometrica: Journal of the Econometric Society*, 245-259 (1945).

21. Sen PK. Estimates of the regression coefficient based on Kendall's tau. *Journal of the American Statistical Association* **63**, 1379-1389 (1968).

22. Thiel H. A rank-invariant method of linear and polynomial regression analysis, Part 3. In: *Proceedings of Koninalijke Nederlandse Akademie van Weinenschatpen A* (ed^(eds) (1950).

23. National Interagency Fire Center. Total Wildland Fires and Acres (1960-2013), **7** <http://www.nifc.gov/fireInfo/fireInfo_stats_totalFires.html> (2014).

24. Canadian National Forestry Database. Forest Fire Statistics by Province/Territory/Agency, 1970-2013, <http://nfdp.ccfm.org/data/compendium/html/comp_31e.html> (2014).

25. Short K. A spatial database of wildfires in the United States, 1992-2011. *Earth System Science Data* **6**, 1-27 (2014).

26. Olson DM, Dinerstein E, Wikramanayake ED, Burgess ND, et al. Terrestrial ecoregions of the world: A new map of life on Earth. *Bioscience* **51**, 933-938 (2001).

27. Le Quéré C*, et al.* Global carbon budget 2013. *Earth Syst Sci Data* **6**, 235-263 (2014).

28. Boden TA, Marland G, Andres RJ. Global, regional, and national fossil-fuel CO_2 emissions. *Carbon Dioxide Information Analysis Center, Oak Ridge National Laboratory, US Department of Energy, Oak Ridge, Tenn, USA doi* **10**, (2009).

29. Houghton RA*, et al.* Carbon emissions from land use and land-cover change. *Biogeosciences* **9**, 5125-5142 (2012).
